# Comparative Efficacy of Different Targeted Therapies in Patients With Moderate‐to‐Severe Ulcerative Colitis: Systematic Review/Network Meta‐Analysis and Mechanistic Overview

**DOI:** 10.1002/prp2.70108

**Published:** 2025-06-05

**Authors:** Youran Dai, Wenhui Yang, Li Xu, Pingting Pan, Shan Liu, Yingzhe Sun, Suying Hu, Qiushuang Li, Fang Hu

**Affiliations:** ^1^ The First Clinical College Zhejiang Chinese Medical University Hangzhou Zhejiang China; ^2^ Department of Anorectal Surgery The First Affiliated Hospital of Zhejiang Chinese Medical University (Zhejiang Provincial Hospital of Traditional Chinese Medicine) Hangzhou Zhejiang China; ^3^ The Second Clinical College Zhejiang Chinese Medical University Hangzhou Zhejiang China; ^4^ Center of Clinical Evaluation and Analysis The First Affiliated Hospital of Zhejiang Chinese Medical University (Zhejiang Provincial Hospital of Chinese Medicine) Hangzhou Zhejiang China; ^5^ The College of Stomatology Zhejiang Chinese Medical University Hangzhou Zhejiang China; ^6^ The school of Pharmacy Zhejiang Chinese Medical University Hangzhou Zhejiang China; ^7^ GCP Clinical Triallnstitution Office The First Affiliated Hospital of Zhejiang Chinese Medical University (Zhejiang Provincial Hospital of Chinese Medicine) Hangzhou China

**Keywords:** comparative efficacy, network meta‐analysis, safety, ulcerative colitis

## Abstract

Ongoing evaluations of targeted therapies for moderate‐to‐severe ulcerative colitis (UC) continue to unfold, with the emergence of novel drugs. However, head‐to‐head trials comparing these therapies are still lacking. The aim of this study is to investigate the therapeutic effects of targeted therapies in moderate‐to‐severe UC. The Cochrane Library, Web of Science, PubMed, and Embase were searched from the inception to November 12, 2024. Statistical analyses included multivariate random effects models and Bayesian modeling. Stratified and sensitivity analyses were also performed. Publication bias was assessed using funnel plots. Outcomes such as clinical response/remission, endoscopic remission, mucosal healing, quality of life, adverse events (AEs), and serious adverse events (SAEs) were used to quantify the relative therapeutic effects. Thirty‐three studies (33 reported on the induction phase; 13 reported on the maintenance phase) were identified. In the induction phase, Upadacitinib 45 mg demonstrated the highest efficacy in achieving clinical remission (OR 10.03; 95% CI, 4.83–20.80), clinical response (OR 7.96; 95% CI, 3.89–16.28), and mucosal healing rate (OR 8.91; 95% CI, 3.36–23.62). Cobitolimod 250 mg was the first‐ranked treatment (SUCRA, 92.67%) in Endoscopic remission. Vedolizumab 108 mg was the best dosage in reducing Adverse Events (AEs). The optimal dosage for reducing Serious Adverse Events (SAEs) was found to be Tulisokibart 1000/500 mg. During the maintenance phase, Etrasimod 2 mg/kg ranked first in clinical remission (OR 9.58; 95% CI, 2.82–32.59), and Upadacitinib 45 mg was superior in endoscopic remission. Additionally, the most effective medication for raising quality of life was Guselkumab 200 mg (OR 3.04; 95% CI, 1.70–5.40). Consequently, there is a need for further high‐quality research to conclusively determine the best therapeutic option.

## Graphical abstract

Efficacy and Safety of Targeted Therapies for Moderate‐to‐Severe Ulcerative Colitis.

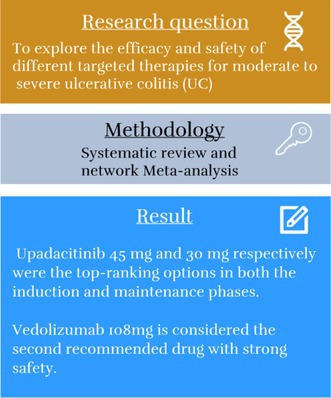

## 1 Introduction

Ulcerative colitis (UC) is a chronic nonspecific intestinal inflammatory disease characterized by inflammatory changes in the colon mucosa. The most common symptoms include diarrhea, hematochezia, and stomach pain. Individuals with UC experience a diminished health‐related quality of life [1] and significant direct and indirect economic burdens [2]. Therefore, it is of great importance to choose an appropriate treatment strategy, especially for patients with moderate‐to‐severe UC.

Conventional therapies for UC include corticosteroids and 5‐aminosalicylates [3]. Over the past decade, the treatment options landscape broadened substantially. This has led to the pursuit of superior treatment goals and more effective strategies, including clinical and endoscopic remission. According to the literature and extensive clinical experience, targeted therapies can be potentially a very effective choice for patients with moderate‐to‐severe UC [4]. As suggested by the name, targeted therapy is an emerging treatment approach that targets specific pathogenic sites or molecular pathways to treat diseases, which has ushered in a novel era in the management of UC. It encompasses a wide array of substances, including antitumor necrosis factor (TNF) antibodies, anti‐integrin antibodies, anti‐interleukin (IL)‐12/23 antibodies, Janus kinase (JAK) inhibitors, and recently also sphingosine 1‐phosphate (S1P) receptor modulators and other emerging therapeutic agents [5]. These treatments represent innovative therapeutic agents that specifically target pro‐inflammatory pathways, thereby regulating and delaying the inflammatory process through modulation of mechanisms like anti‐inflammatory signaling and lymphocyte adhesion/migration.

To our knowledge, at the time of submission there are few head‐to‐head comparisons of these targeted therapies. To address this gap, network meta‐analysis (NMAs) can be a useful tool to help inform treatment decisions as the number of targeted therapies for moderate‐to‐severe UC keeps expanding. Although numerous NMAs have investigated targeted therapies for severe UC, we felt that the continual emergence of new targeted therapeutics has created a gap in the literature. Therefore, we not only comprehensively evaluated the effectiveness and safety of current targeted therapeutic drugs but also paid special attention to the newly developed drugs. Additionally, we conducted an overview on the mechanisms of various targeted therapeutic drugs. Through our analysis, we sought to identify the most effective drug and dosage regimen for patients during both the induction and remission phases of their treatment, aiming to provide clinicians and patients with more comprehensive, cutting‐edge treatment options.

## 2 Methods

The protocol for this systematic review and NMA was registered in PROSPERO (CRD42023473114). This analysis adhered to the guidelines in the Preferred Reporting Items for Systematic Reviews and Meta‐Analyses (PRISMA) extended statement [6].

### 2.1 Search Strategy

We conducted a comprehensive search across various electronic databases (Embase, PubMed, Web of Science, and the Cochrane Library), from their inception to November 12, 2024, using predefined terms. Clinical trials, reference lists, and relevant conference proceedings (i.e., Cochrane trials, American College of Gastroenterology Annual Meeting, and Advances in IBD Meeting) were further searched to identify additional eligible studies. The search utilized a range of MeSH terms related to “ulcerative colitis”, “Infliximab”, “Adalimumab”, “Golimumab”, “Ustekinumab”, “Gueslkumab”, “Basiliximab”, “Vedolizumab”, “Cobitolimod”, “Eldelumab”, “Visilizumab”, “Daclizumab”, “Etrolizumab”, “PF‐00547659”, “BMS‐936557”, “Tofacitinib”, “Filgotinib”, “Upadacitinib”, “Ozanimod”, “Etrasimod”, “Apremilast”, “AJM‐300”, “Obefazimod”, “Ivarmacitinib”, “Izencitinib”, “Fingolimod”, “Amiselimod”, “Mongersen”, “Amiselimod”, “Risankizumab”, “GSK‐2982772”, “Brazikumab”, “Tulisokibart”, and “randomized controlled trial”. The full search strategies for each database are included in Table S1.

### 2.2 Selection Criteria

Targeted therapy refers to the blockage or intervention in established pathways to regulate and treat immune‐mediated disorders. The rationale of targeted therapy has been initially explored in rheumatic and dermatological diseases. Targeted therapy regimens for UC patients included antitumor necrosis factor (TNF) antibodies, anti‐integrin antibodies, anti‐interleukin (IL)‐12/23 antibodies, Janus kinase (JAK) inhibitors, and recently also sphingosine‐1‐phosphate (S‐1‐P) receptor modulators and so on [7, 8, 9]. Two researchers independently screened the literature. In case of any disparities in outcomes, the final decision was reached through discussion. Our study included only randomized control trials involving individuals with moderately to severely UC undergoing targeted therapies. Eligible candidates were patients above 18 years at the time of the screening phase, of any gender, with a diagnosis of moderate‐to‐severe ulcerative colitis with endoscopic and histopathologic evidence. Disease flare was defined as a Mayo score of ≥ 4 with a Mayo endoscopic sub score ≥ 2 within 2 weeks before study drug administration. They should have been previously untreated or on stable doses of 5‐ASA, corticosteroids, AZA, and/or 6‐MP. Patients with moderate‐to‐severe ulcerative colitis refractory to steroids or glucocorticoids were excluded. The control group was defined as either a placebo or other targeted therapies, which were used as a comparative measure. This systematic review and network meta‐analysis of randomized controlled trials (RCTs) focused on evaluating the efficacy and safety of the treatment regimens. Any studies, reviews, letters, editorials, conference abstracts, notes, and repeatedly published literature with incomplete data were excluded. There were no restrictions on the language, nationality, publication date, or publication status of the studies.

### 2.3 Primary and Secondary Outcomes

Mayo clinical score (MCS) is routinely utilized to categorize UC and is the main efficacy measure for HTA economic analysis [10]. Efficacy outcomes of interest were clinical response (total MCS score ≤ 2 and no individual sub score > 1), clinical remission (≥ 30% and decrease ≥ 3 points from baseline MCS and rectal bleeding sub score of 0–1), endoscopic remission (MCS endoscopic sub score ≤ 1), mucosal healing (MCS endoscopic score of 0 and a Geboes score < 2), Quality of life (Inflammatory Bowel Disease Questionnaire [IBDQ] score increase ≥ 16) during the induction or maintenance phase, as defined in the studies. Primary outcomes included specific rates of clinical remission and adverse events (AEs). Secondary outcomes included pooled rates of clinical response, endoscopic remission, endoscopic response, mucosal healing, serious adverse events (SAEs), and quality of life. We collected data on the percentage of patients who met each of the efficacy outcomes.

### 2.4 Data Extraction

Two authors independently conducted a thorough elimination of all duplicated articles using EndNote X9. All studies were then screened according to the inclusion and exclusion criteria. Disagreements were resolved by a third author. In the case of multiple reports of the same trial, the most up‐to‐date report was included. Detailed data were extracted by two independent reviewers from selected clinical trials into a Microsoft Excel spreadsheet. The following information was extracted: author, publication year, sample size, baseline patient characteristics, methods of treatment and specific doses in each arm, median follow‐up, and efficacy for outcome indicators. When available, intention‐to‐treat (ITT) analyses were extracted. If ITT data were not provided, we utilized the data reported by the authors. Data reported at the end of treatment were extracted unless unavailable. Supplement materials of the literature, conference papers, post hoc analyses, etc. with the same NCT sequence/author were further searched to obtain complete outcome data. If two randomized controlled trials (RCT) were presented in one paper, we combined data from patients with comparable baseline characteristics and the same treatment regimen. Our study aimed to identify the most effective treatment regimen and optimal dosage for moderate‐to‐severe UC patients. To ensure accurate outcome indicator efficacy, we established specific inclusion criteria. We only considered outcome data from the best treatment identified in each trial. The therapeutic dose should be consistent with guidelines and expert consensus recommendations, which are widely utilized in clinical practice. If multiple treatment measures showed no significant difference in curative effect, we excluded the outcome data of the treatment with a lower clinical response rate, based on the value of the clinical response rate.

### 2.5 Quality Assessment

The Cochrane framework ROB2.0, as outlined in the Cochrane Handbook for Systematic Reviews of Interventions, was used to assess the quality of the included studies [11]. This comprehensive framework assesses various aspects such as random sequence generation, allocation concealment, measurement of outcome, and blinding. The risk of bias is categorized into three levels: Low risk, some concerns, and high risk. Two investigators conducted an independent summary of the quality assessment and sought guidance from the supervisor in case of any disagreements.

### 2.6 Statistical Analysis

Based on the frequentist approach, we performed a NMA to compare the effectiveness of each treatment by integrating all available study results. In contrast to traditional meta‐analyses, our approach allowed for indirect comparisons of treatments based on common comparator groups. Firstly, we utilized the netmeta package in R software version 4.3.0 to estimate the therapeutic effects of each direct pairwise comparison of drugs, employing a multivariate random effects model and restricted maximum likelihood estimation [12]. Empirical and simulation studies have demonstrated that, in most cases, frequentist and Bayesian methods can yield overlapping results in network meta‐analysis [13]. The frequentist approach, being independent of prior distributions, is considered more objective and unbiased. Additionally, we also employed the getmtc package in R software version 4.3.0 to estimate the Surface Under the Cumulative Ranking Curve (SUCRA) and ranking probabilities through Bayesian modeling [14]. This package utilizes the Markov Chain Monte Carlo method, with 5000 iterations and 20 000 parameter estimates during the iterative process. The SUCRA direction was set to 1, where a higher SUCRA score indicates better therapeutic efficacy for effectiveness indicators, and a lower score suggests better safety for safety indicators. Since the efficacy and safety endpoints were bivariate variables (ratio [%]), odds ratios (OR), and 95% confidence intervals (CI) were used to analyze the results. Additionally, we explored the presence of publication bias by creating comparison‐adjusted funnel plots using the metafor package.

## 3 Results

### 3.1 Search Results

**FIGURE 1:**
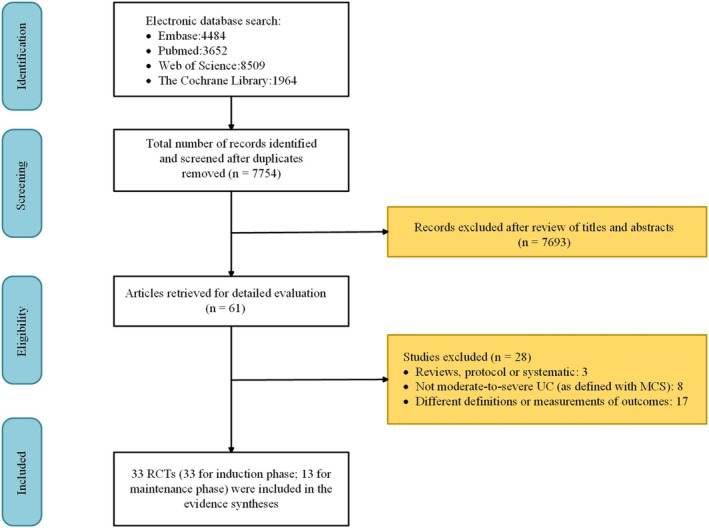
Search strategy for targeted therapy in moderate‐to‐severe ulcerative colitis.

Using the search strategy described above, 18,609 articles were retrieved. After the removal of duplicates, a total of 7754 articles remained. Among them, 61 full‐text articles were examined for eligibility qualifications. Eventually, a total of 33 RCT studies met our inclusion criteria and were included in the NMA, with a total of 13,564 patients [15, 16, 17, 18, 19, 20, 21, 22, 23, 24, 25, 26, 27, 28, 29, 30, 31, 32, 33, 34, 35, 36, 37, 38, 39, 40, 41, 42, 43, 44, 45, 46, 47]. All included trials reported the results of the induction phase. Fourteen trials reported outcomes in the maintenance phase. Figure 1 shows the PRISMA diagram, and Table 1 summarizes the baseline characteristics and efficacy outcomes for the included studies.

**TABLE 1:** Trial and patient characteristics in included trials of induction and maintenance therapy for moderate–severe ulcerative colitis.

Author, year	Disease stage	Mayo scores	Treatment duration (induction phase)	Treatment duration (maintenance phase)	Sex (M/F)	Number of participants	Randomized treatments	Age (mean ± SD), years
Raja Atreya, 2020	Moderately to severely active ulcerative colitis (left side)	6–12	6 weeks	/	59/27	86	Cobitolimod 250 mg	46.2 ± 14.0
							PBO	45.5 ± 15.2
Walter Reinisch, 2011	Moderately to severely active ulcerative colitis	6–12	8 weeks	/	160/100	260	Adalimumab 160/80 group	37.4 ± 11.0
							PBO	37.8 ± 10.4
W. J. Sandborn, 2013	Moderately to severely active ulcerative colitis	6–12	8 week	52 weeks	294/200	494	Adalimumab 160/80 group	39.6 ± 12.5
							PBO	41.3 ± 13.2
P. Rutgeerts, 2015	Moderately to severely active ulcerative colitis	6–12	6 weeks	/	83/49	132	Golimumab2 mg/kg	42.3 ± 13.1
							PBO	40.9 ± 12.6
Silvio Danese, 2022	Moderately to severely active ulcerative colitis (naive to tumor necrosis factor inhibitors)	5–9	10 weeks	54 weeks	250/147	397	Etrolizumab 105 mg/kg	37.7 ± 10.6
							Infliximab 5 mg/kg	37.5 ± 9.5
Bruce E. Sands, 2019	Moderately to severely active ulcerative colitis	6–12	14 weeks	52 weeks	450/321	771	Vedolizumab 300 mg	40.8 ± 13.7
							ADA 160/80/40	40.5 ± 13.4
Silvio Danese, 2022	Moderately to severely active ulcerative colitis	5–9	8 weeks	52 weeks	616/372	988	Upadacitinib 45 mg	41.5 ± 23.5
							PBO	43.2 ± 23.5
Séverine Vermeire, 2021	Moderately to severely active ulcerative colitis	4–9	12 weeks	52 weeks	46/28	74	Etrasimod 2 mg/kg	39.2 ± 11.0
							PBO	46.2 ± 15.1
William J. Sandborn, 2020	Moderately to severely active ulcerative colitis	5–9	8 weeks	/	66/36	102	Upadacitinib 45 mg	38.6 ± 12.0
							PBO	40.7 ± 10.4
William J. Sandborn, 2014	Moderately to severely active ulcerative colitis	6–12	12 weeks	54 weeks	164/146	310	Golimumab 2 mg/kg	39.1 ± 13.1
							PBO	40.2 ± 14.1
William J. Sandborn, 2016	Moderately to severely active ulcerative colitis	6–12	11 week	/	87/81	168	Eldelumab 25 mg/kg	39.0 ± 12.7
							PBO	42.7 ± 14.2
Makoto Naganuma, 2022	Moderately to severely active ulcerative colitis (on stable doses of 5‐ASA, corticosteroids, AZA and/or 6‐MP)	6–12	10 weeks	60 weeks	99/65	164	Vedolizumab 300 mg	42.3 ± 14.2
							PBO	42.4 ± 14.7
Brian G Feagan, 2021	Moderately to severely active ulcerative colitis	6–12	11 weeks	58 weeks	444/342	786	Filgotinib 200 mg	42.5 ± 13.8
							PBO	42.5 ± 13.9
William J Sandborn, 2023	Moderately to severely active ulcerative colitis (history of inadequate response or loss or intolerance of at least one therapy)	4–9	12 weeks	52 weeks	240/193	433	Etrasimod 2 mg/kg	41.2 ± 14.0
							PBO	38.9 ± 14.0
Severine Vermeire, 2022	Moderately to severely active ulcerative colitis	≥ 5	16 weeks	48 weeks	67/60	127	Obefazimod 50 mg	40.2 ± 13.9
							PBO	41.1 ± 14.4
David T Rubin, 2022	Moderately to severely active ulcerative colitis (naive to tumor necrosis factor inhibitors)	6–12	10 weeks	/	239/189	428	Etrolizumab 105 mg	38.7 ± 11.4
							PBO	37.6 ± 11.5
Lloyd Mayer, 2013	Moderately to severely active ulcerative colitis (on stable doses of 5‐ASA, corticosteroids, AZA and/or 6‐MP)	6–10	8 weeks	/	68/41	109	Eldelumab 10 mg/kg	44.7 ± 12.8
							PBO	41.8 ± 14.2
Laurent Peyrin‐Biroulet, 2023	Moderately to severely active ulcerative colitis	5–9	12 weeks	/	126/80	206	Guselkumab 200 mg	43.3 ± 14.28
							PBO	41.2 ± 15.05
Paul Rutgeerts, 2005	Moderately to severely active ulcerative colitis	6–12	8 weeks	54 weeks	150/92	242	Infliximab 5 mg/kg	42.4 ± 14.3
							PBO	41.4 ± 13.7
Severine Vermeire, 2022	Moderately to severely active ulcerative colitis (not have received previous anti‐TNF treatment, must have had an inadequate response, loss of response, or intolerance to previous immunosuppressant or corticosteroid treatment, or both)	6–12	10 weeks	62 weeks	112/102	214	Etrolizumab 105 mg	38.6 ± 10.1
							PBO	39.0 ± 11.7
William J. Sandborn, 2020	Moderately to severely active ulcerative colitis (history of inadequate response or loss or intolerance of at least one therapy)	6–12	6 weeks	52 weeks	99/63	162	Vedolizumab 108 mg	38.1 ± 13.1
							PBO	39.4 ± 11.7
Baili Chen, 2022	Moderately to severely active ulcerative colitis	5–9	8 weeks	/	45/37	82	Ivarmacitinib 4 mg	39.6 ± 10.0
							PBO	42.7 ± 12.9
Brian G. Feagan, 2013	Moderately to severely active ulcerative colitis	6–12	6 weeks	52 weeks	525/881	1406	Vedolizumab 300 mg	40.1 ± 13.2
							PBO	41.2 ± 12.5
Bruce E. Sands, 2024	Moderately to severely active ulcerative colitis (exposed to conventional therapies but were naive to ATs)	6–12	10 weeks	52 weeks	254/179	424	Ozanimod 0.92 mg	41.8 ± 13.3
							PBO	42.7 ± 13.8
Geert D'Haens, 2023	Moderately to severely active ulcerative colitis	4–9	12 weeks	52 weeks	695/467	1162	Mirikizumab 300 mg	42.9 ± 13.9
							PBO	41.3 ± 13.8
William J. Sandborn, 2017	Moderately to severely active ulcerative colitis	6–12	8 weeks	52 weeks	668/471	1139	Tofacitinib 10 mg	41.8 ± 15.3
							PBO	41.3 ± 14.1
B.E. Sands, 2019	Moderately to severely active ulcerative colitis	6–12	8 weeks	44 weeks	392/249	641	Ustekinumab 6 mg/kg	41.7 ± 13.7
							PBO	41.2 ± 13.5
Edouard Louis, 2024	Moderately to severely active ulcerative colitis	6–12	8 weeks	52 weeks	592/383	975	Risankizumab 1200 mg	41.7 ± 13.7
							PBO	42.9 ± 13.9
G Van Assche, 2006	Moderately to severely active ulcerative colitis	6–12	8 weeks	/	58/45	103	Daclizumab 2 mg/kg	40.7 ± 13.2
							PBO	42.6 ± 15.4
Bruce E. Sands, 2024	Moderately to severely active ulcerative colitis	6–12	12 weeks	/	96/114	210	Tulisokibart 1000/500 mg	42.2 ± 16.3
							PBO	40.4 ± 14.4
Séverine Vermeire, 2017	Moderately to severely active ulcerative colitis	6–12	12 weeks	/	129/14	143	PF‐00547659 22.5 mg	42.1 ± 14.7
							PBO	38.6 ± 12.7
Katsuyoshi Matsuoka, 2022	Moderately to severely active ulcerative colitis	6–12	8 weeks	/	123/80	203	AJM‐300960 mg	44.0 ± 14.2
							PBO	42.8 ± 13.3
Silvio Danese, 2022	Moderately to severely active ulcerative colitis	6–12	8 weeks	/	388/253	641	Ustekinumab 6 mg/kg	41.7 ± 13.7
							PBO	

### 3.2 Characteristics of the Studies

Baseline characteristics, including age, sex, and weight, were similar across trials (Table 1). The mean age range was 37.4–46.2 years, and 43.98% of patients were female. At the initiation of targeted therapy, 6.08% of patients were treatment‐naive to tumor necrosis factor inhibitors, and 9.09% of patients had a history of inadequate response or loss or intolerance to at least one therapy. Thirty‐three RCTs included reported data on the induction period, with 14 of them also reporting data on the maintenance period. All trials included in the study reported clinical response rates and projected rates of adverse events. Twenty‐eight trials reported clinical response rates, while 15 presented endoscopic response rates, 17 reported mucosal healing rates, and nine reported quality‐of‐life results. Additionally, a total of 30 trials covered Serious Adverse Events (SAEs) as a result. The Cochrane assessment of within‐trial bias revealed 20 (57.60%) studies with a low risk of bias, 11 (33.33%) studies with some risk, and 2 (6.10%) trials with a high risk. The risk of bias assessment was shown in Table S2 and Figure S1. There was no evidence of publication bias with funnel plot analysis in Figure S5.

### 3.3 NMA of the Efficacy of Different Targeted Therapies in RCTs

This study involved a comparison of different targeted therapies. The nodes in the processing network for each antibody were sized proportionally to the number of random participants, while the thickness of each line in the network diagram was proportional to the number of lines in the network. Detailed information on this NMA is presented in Figure S2.

### 3.4 Induction Phase Analyses

#### 3.4.1 Clinical Remission

**FIGURE 2:**
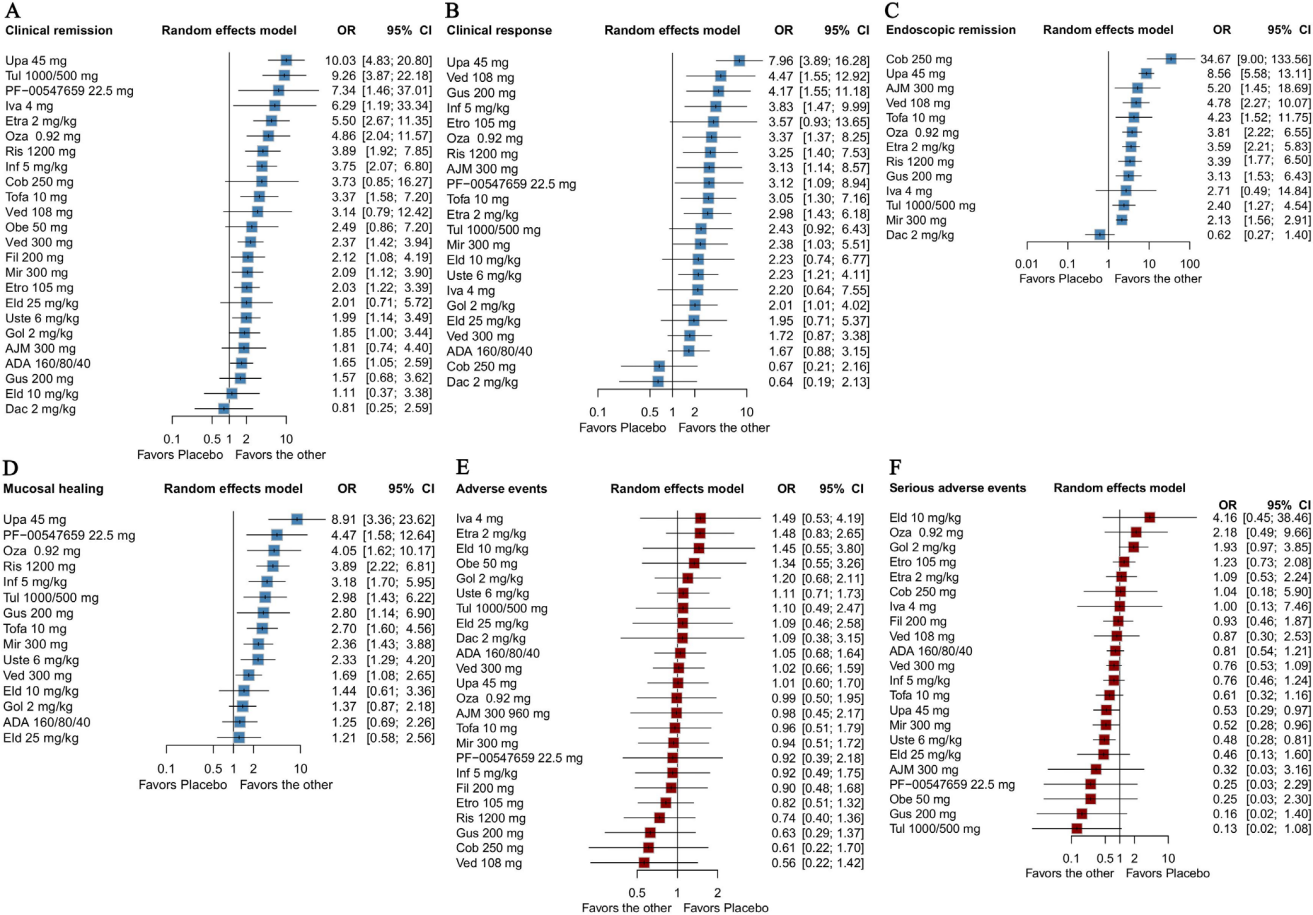
Main efficacy and safety outcomes (i.e., clinical remission, clinical response, endoscopic remission, and mucosal healing in Induction phase; AEs; SAEs) of targeted therapies in moderate‐to‐severe ulcerative colitis patients: Forest plot. PBO: Placebo; ADA 160/80/40 group: ADA 160/80/40; Cobitolimod 250 mg: Cob 250 mg; Eldelumab 25 mg/kg: Eld 25 mg/kg; Eldelumab 10 mg/kg: Eld 10 mg/kg; Etrasimod 2 mg/kg: Etra 2 mg/kg; Etrolizumab 105 mg: Etro 105 mg; Filgotinib 200 mg: Fil 200 mg; Golimumab 2 mg/kg: Gol 2 mg/kg; Guselkumab 200 mg: Gus 200 mg; Infliximab 5 mg/kg: Inf 5 mg/kg; Obefazimod 50 mg: Obe 50 mg; Upadacitinib 45 mg: Upa 45 mg; Vedolizumab 108 mg: Ved 108 mg; Vedolizumab 300 mg: Ved 300 mg; Ivarmacitinib 4 mg: Iva 4 mg; Ozanimod 0.92 mg: Oza 0.92 mg; Tofacitinib 10 mg: Tofa 10 mg; Ustekinumab 6 mg/kg: Uste 6 mg/kg; Mirikizumab 300 mg: Mir 300 mg; Risankizumab 1200 mg: Ris 1200 mg; Daclizumab 2 mg/kg: Dac 2 mg/kg; Tulisokibart 1000/500 mg: Tul1000/500 mg; PF‐00547659 22.5 mg: PF‐00547659 22.5 mg; AJM 300960 mg: AJM 300960 mg; CI: confidence intervals; OR, odd ratios.

All agents were superior to placebo for the induction of clinical remission (Figure 2A and Table S3A), except Daclizumab 2 mg/kg. The effect size was strongest for Upadacitinib 45 mg (OR 10.03; 95% CI, 4.83–20.80). The SUCRA indicated a 92.46% probability that Upadacitinib 45 mg is the preferred treatment in enhancing clinical remission. The second‐ranked treatment was Tulisokibart 1000/500 mg (SUCRA, 88.18%), followed by PF‐00547659 22.5 mg (SUCRA, 83.24%), Etrasimod 2 mg/kg (SUCRA, 78.50%), and Ivarmacitinib 4 mg g (SUCRA, 78.38%). The integrity ranking results are presented in Table 2.

**TABLE 2:** SUCRA values and ranks of treatments (i.e., clinical remission, clinical response, endoscopic remission, and mucosal healing in induction phase; AEs; SAEs).

Treatment	Clinical remission		Clinical response		Endoscopic remission		Mucosal healing		AEs		SAEs	
	SUCRA	Rank	SUCRA	Rank	SUCRA	Rank	SUCRA	Rank	SUCRA	Rank	SUCRA	Rank
Placebo	8.02	25	11.18	21	14.00	12	12.82	16	50.69	13	68.00	6
ADA 160/80/40 group	27.58	21	30.74	20	/	/	24.65	14	54.99	10	53.36	11
Cobitolimod 250 mg	63.78	9	8.91	22	92.67	/	24.39	15	22.88	23	62.22	8
Eldelumab 25 mg/kg	38.82	16	39.85	18	/	/	31.13	12	56.39	8	33.81	17
Eldelumab 10 mg/kg	18.02	23	46.17	15	/	/	/	/	72.99	3	93.10	1
Etrasimod 2 mg/kg	78.50	4	59.52	11	53.38	5	/	/	78.11	1	68.47	5
Etrolizumab 105 mg	37.40	17	64.45	6	/	/	/	/	33.78	21	75.18	4
Filgotinib 200 mg	40.12	14	/	/	/	/	/	/	42.44	20	65.21	7
Golimumab 2 mg/kg	32.87	20	39.98	17	/	/	26.43	13	73.88	2	87.19	3
Guselkumab 200 mg	27.23	22	72.89	3	46.65	8	58.13	7	21.17	24	12.24	22
Infliximab 5 mg/kg	64.95	8	69.69	4	/	/	63.02	5	43.90	19	51.65	12
Obefazimod 50 mg	47.81	12	/	/	/	/	/	/	68.24	5	19.15	21
Upadacitinib 45 mg	92.46	1	93.30	1	80.68	1	90.11	1	51.97	12	36.00	16
Vedolizumab 108 mg	58.72	10	75.04	2	59.76	3	/	/	17.93	25	58.42	10
Vedolizumab 300 mg	45.52	13	32.43	19	/	/	34.95	11	52.41	11	51.35	13
Ivarmacitinib 4 mg	78.38	5	46.39	14	46.51	9	/	/	72.43	4	59.12	9
Ozanimod 0.92 mg	73.13	6	64.49	5	52.57	6	72.11	3	49.64	14	87.21	2
Tofacitinib 10 mg	50.01	11	59.74	10	58.36	4	56.41	8	47.15	16	44.28	14
Ustekinumab 6 mg/kg	36.77	18	44.51	16	/	/	50.25	10	59.37	6	31.72	18
Mirikizumab 300 mg	10.26	24	8.53	23	10.64	13	/	/	55.44	9	/	/
Risankizumab 1200 mg	38.93	15	48.22	13	33.97	11	50.37	9	45.32	17	36.29	15
Daclizumab 2 mg/kg	65.67	7	62.93	7	49.43	7	70.29	4	27.87	22	/	/
Tulisokibart 1000/500 mg	88.18	2	49.22	12	38.41	10	60.44	6	56.85	7	9.80	23
PF‐00547659 22.5 mg	83.24	3	60.86	9	/	/	74.52	2	45.24	18	20.59	20
AJM 300960 mg	33.62	19	60.95	8	62.97	2	/	/	49.18	15	25.64	19

#### 3.4.2 Clinical Response

Compared with PBO, Cobitolimod 250 mg and Daclizumab 2 mg/kg did not demonstrate any advantages in improving the clinical response rate (Figure 2B and Table S3B). Among them, the effect size was strongest for Upadacitinib 45 mg (OR 7.96; 95% CI, 3.89–16.28; SUCRA 93.30%), closely followed by Vedolizumab 108 mg (SUCRA, 75.04%), Guselkumab 200 mg (SUCRA, 72.89%) in the third position, then Infliximab 5 mg/kg (SUCRA, 69.69%), and finally Ozanimod 0.92 mg (SUCRA, 64.49%). The ranking results are presented in Table 2.

#### 3.4.3 Endoscopic Remission

All treatment measures were superior to PBO, and most of them showed statistical significance (Figure 2C and Table S3C). However, 2 mg/kg Daclizumab did not show any improvement. The SUCRA result illustrated that Cobitolimod 250 mg was the first‐ranked treatment (SUCRA, 92.67%), followed by Upadacitinib 45 mg (SUCRA, 80.68%), AJM 300960 mg (SUCRA, 62.97%), Vedolizumab 108 mg (SUCRA, 59.76%), and finally Tofacitinib 10 mg (SUCRA, 58.36%). The ranking results are presented in Table 2.

#### 3.4.4 Mucosal Healing

All agents were superior to placebo for induction of clinical remission, and the effect size was strongest for Upadacitinib 45 mg (OR 8.91; 95% CI, 3.36–23.62). Specific data are shown in Figure 2D and Table S3D. According to the SUCRA result (Table 2), Upadacitinib 45 mg showed superior efficiency (SUCRA,90.11%). Followed by PF‐00547659 22.5 mg (SUCRA, 74.52%), Ozanimod 0.92 mg (SUCRA, 72.11%), Daclizumab 2 mg/kg (SUCRA, 70.29%), and Guselkumab 200 mg (SUCRA, 40.55%).

### 3.5 Maintenance Phases Analyses

#### 3.5.1 Clinical Remission

A total of 13 studies provided clinical remission rates for the maintenance phases, and all interventions were found to be superior to placebo with a significant statistical difference (Figure S3A). Etrasimod 2 mg/kg was the optimal treatment (OR 9.58; 95% CI, 2.82–32.59; SUCRA, 88.27%). The remaining top five treatments were Upadacitinib 30 mg (SUCRA, 84.53%), Vedolizumab 108 mg (SUCRA, 73.23%), Vedolizumab 300 mg (SUCRA, 62.17%), and Mirikizumab 200 mg (SUCRA, 53.90%). The NMA results and ranking results are presented in Tables S3E and S4A.

#### 3.5.2 Endoscopic Remission

Six studies reported data on endoscopic response rates during maintenance phases. All included treatments were more effective compared to the placebo, while no significant statistical differences were found (Figure S3B and Table S3F). The SUCRA result revealed that Upadacitinib 30 mg was the top‐ranked therapeutic measure (SUCRA 82.11%), followed by Etrasimod 2 mg/kg (SUCRA 55.34%), Infliximab 5 mg/kg (SUCRA 52.77%), Ustekinumab 90 mg (SUCRA 51.55%), and Risankizumab 180 mg (SUCRA 48.13%). The ranking results are presented in Table S4A.

### 3.6 Safety Outcomes

#### 3.6.1 Adverse Events

In terms of AEs, most treatment groups demonstrated a reduction in the incidence of adverse events compared with PBO (Figure 2E). Following the SUCRA ranking result, the highest‐risk therapy is Etrasimod 2 mg/kg (SUCRA, 78.11%), Golimumab 2 mg/kg (SUCRA, 73.88%), Eldelumab 10 mg/kg (SUCRA, 72.99%), Ivarmacitinib 4 mg (SUCRA, 72.43%), and Obefazimod 50 mg (SUCRA,68.24%). The NMA results and ranking results are presented in Table 2 and Table S3G.

#### 3.6.2 Serious Adverse Events

Seven interventions (Eldelumab 10 mg/kg, Ozanimod 0.92 mg, Golimumab 2 mg/kg, Etrolizumab 105 mg, Etrasimod 2 mg/kg, Cobitolimod 250 mg, and Ivarmacitinib 4 mg) may increase the rate of SAEs, while other drugs demonstrate a reduction in the incidence of SAEs. Among them, Tulisokibart 1000/500 mg was the optimal drug (Figure 2F). We used the same method as AES for SUCRA ranking; the drug with the highest risk is Eldelumab 10 mg/kg (SUCRA, 93.10%), Ozanimod 0.92 mg (SUCRA, 87.21%), Golimumab 2 mg/kg (SUCRA, 87.19%), Etrolizumab 105 mg (SUCRA, 75.18%), and Etrasimod 2 mg/kg (SUCRA,68.47%). The NMA results and ranking results are presented in Table 2 and Table S3H.

### 3.7 Quality of Life

A total of seven studies presented quality‐of‐life assessment data. A study by Sands et al. (2019) was removed from the network analysis because it could not be netted with the other groups [42]. Ultimately, six studies were selected for quality‐of‐life analysis. The vast majority of naturopathic measures showed superiority compared with placebo, with no statistically significant differences (Table S3I and Figure S4). Among them, Guselkumab 200 mg improvement was equivalent to placebo (OR 3.04; 95% CI, 1.70–5.44; SUCRA 75.66%). Followed by Filgotinib 200 mg (SUCRA 61.93%), Cobitolimod 250 mg (SUCRA 59.28%), Upadacitinib 45 mg (SUCRA 53.84%), and Eldelumab 25 mg/kg (SUCRA 48.79%). The ranking results are presented in Table S4B.

## 4 Discussion

Currently, studies which directly compare the effectiveness of various targeted therapies for moderate‐to‐severe UC are still limited. As the number of available treatments increases, clinicians confront a more intricate decision‐making process, hindered by lengthy and limited face‐to‐face trial cycles. Network meta‐analyses have offered some insights; for instance, Singh et al. found Infliximab to be the most effective biological therapy in terms of clinical remission and endoscopic improvement [34]. Meanwhile, Lasa JS et al. indicated that Upadacitinib was superior in inducing clinical remission, while Vedolizumab exhibited the best safety profile [35]. Additionally, Burr, NE et al. revealed that Upadacitinib 45 mg was the most effective agent for clinical remission, whereas Infliximab 10 mg/kg led in endoscopic improvement [36]. Although these studies provide some reference basis for clinical decision‐making [48, 49, 50], they do not fully encompass all targeted therapies. With the continuous emergence of new targeted drugs, a comprehensive assessment of their effectiveness and safety is particularly crucial. This NMA analysis is performed on a variety of targeted therapies, including newly developed drugs, stratified by treatment stage (induction and maintenance) and taking into account the specific dose of each drug.

UC usually refers to diffuse superficial mucosal inflammation limited to the colon, extending from the rectum to the proximal end of the colon [51]. The current mainstream theory is the inflammation pathogenesis theory. Inflammation damages mucosal integrity and tight junctions, infiltrating a significant number of immune cells, causing overexpression of particular receptors and cell adhesion molecules [52, 53]. Due to multiple potential pathological factors, the complex immune response of the body provides numerous therapeutic targets [54]. The targeted therapy for UC is achieved by modulating downstream inflammation through the blockade of molecular pathways and signal transduction. The mechanisms of action for these various drugs are summarized in Table 3 [5, 14, 50, 55, 56, 57, 58, 59, 60, 61, 62, 63, 64, 65, 66, 67, 68, 69, 70, 71, 72].

**TABLE 3:** Categorization of targeted therapeutics and overview of mechanisms of action.

Type	Drugs	Overview of the mechanism	Therapeutic target
Anti‐IL‐12/IL‐23 inhibitor	Ustekinumab	Suppressing the IL‐12/23 axis to mitigate excessive Th1/Th17 immune responses	p40
	Brazikumab		p19
	Gueslkumab		p19
JAK inhibitors	Tofacitinib	JAK inhibitors can target and suppress cytokines such as IL‐2, IL‐6, IL‐12, IL‐21, IL‐23, or interferon (IFN)‐γ, thereby improving intestinal inflammation	JAK1, JAK2, JAK3
	Filgotinib		JAK1
	Upadacitinib		JAK1
	Ivarmacitinib		JAK1
	Izencitinib		JAK1, JAK2, JAK3, TYK2
TNF antibodies	Infliximab	Regulate immune system function by inhibiting the biological activity of TNF	TNF‐α
	Adalimumab		TNF‐α
	Golimumab		TNF‐α
	GSK2982772		TNF‐α
Integrin	AJM300	Block the binding of integrins to the extracellular matrix, inhibit their signal transduction, and induce endothelial cell apoptosis	α4β7; α4β1
	Vedolizumab		α4β7; MAdCAM‐1
	Etrolizumab		α4β7; αEβ7
	PF‐00547659		MAdCAM‐1
S1P receptor modulators	Fingolimod	Block the transmission of the S1P signaling pathway to regulate immune responses	S1P1–5
	Amiselimod		S1P1; S1P5
	Etrasimod		S1PR1; S1PR4; S1PR5
	Ozanimod		S1P1; S1P5
IL‐2R inhibitor	Basiliximab	Inhibit IL‐2 signaling to regulate the homeostasis and function of regulatory T cells (Tregs).	IL‐2Rα
	Daclizumab		IL‐2Rα
CXCL‐10/IP‐10 antibody	Eldelumab	Inhibit the chemotaxis, proliferation, and inflammatory response of immune cells mediated by CXCL‐10/IP‐10.	CXCL‐10/IP‐10
	BMS‐936557		CXCL‐10/IP‐10
SMAD7 inhibitor	Mongersen	Promote the degradation of Smad7 to restore the anti‐inflammatory effects of TGF‐β1	Smad7 mRNA
PDE4 inhibitor	Apremilast	Inhibit cAMP levels and down‐regulate the release of pro‐inflammatory cytokines	PDE4
Anti‐CD3 antibody	Visilizumab	Induction of T cell tolerance and production of regulatory T cells (TREGs)	CD3
RT inhibitors	Obefazimod	Induce the expression of a single microRNA (miRNA) product, miR‐124	CBC
Anti‐TL1A monoclonal antibody	Tulisokibart	Specifically bind to TL1A, blocking the interaction between TL1A and its receptor DR3	TNFSF15/TL1A

Abbreviations: CXCL‐10, C‐X‐C motif chemokine ligand 10; IL, interleukin; JAK, janus kinases; PDE4, phosphodiesterase 4; RT, nucleoside reverse transcriptase; S1P, sphingosine‐1‐phosphate; TL1A/TNFSF15, tumor necrosis factor‐like cytokine 1A/tumor necrosis factor superfamily member 15; TNF, tumor necrosis factor.

The results of this network meta‐analysis demonstrated that most targeted therapies were superior to placebo. Taking into account the effectiveness indicators comprehensively, we hypothesize that Upadacitinib at a dosage of 45 mg is the optimal choice for the induction phase. Meanwhile, both Upadacitinib at 30 mg and Etrasimod at 2 mg/kg are the best options for the maintenance phase. In terms of safety, Vedolizumab at a dosage of 108 mg demonstrates superior performance. However, for reducing severe adverse reactions, Tulisokibart at a dosage of 1000/500 mg is also a commendable choice. The most effective medication for raising quality of life was Guselkumab 200 mg. Mohammad Shehab's study also yielded this result, which reflects the reliability of our analysis results [73].

Upadacitinib is a second‐generation JAK inhibitor. In March 2022, Upadacitinib was approved by the FDA for the treatment of moderately to severely active UC at an induction dose of 45 mg/day for 8 weeks and a maintenance dose of 15 or 30 mg/day [74]. The conclusions of our study align fully with the FDA treatment standards, underscoring the scientific and guiding significance of our research; furthermore, they are aligned with the conclusions drawn by other researchers. Currently, the recommended medications for the treatment of moderate‐to‐severe UC in the guidelines include systemic corticosteroids, TNF inhibitors, Vedolizumab, Tofacitinib, and Ustekinumab. Upadacitinib is not listed as a first‐line recommended medication in the guidelines [3, 75]. Based on our conclusions and those from previous studies, we believe that Upadacitinib has the potential to become a preferred treatment option for inducing remission in patients with moderate‐to‐severe UC in the future. There is a need for further research to establish its position in treatment protocols.

Tulisokibart represents a novel targeted therapy. Our research has demonstrated that Tulisokibart exhibits favorable safety profiles. Moreover, it shows significant advantages in inducing clinical remission (OR 9.26; 95% CI, 3.87–22.18; SUCRA 88.18%). In patients with genetic sensitivity, the clinical remission rate for Tulisokibart is markedly higher than that of the placebo group (26% vs. 1%; *p* < 0.001) [41]. Consequently, we believe that Tulisokibart offers a new therapeutic option for patients with ulcerative colitis and holds promise as one of the key tools in personalized medicine in the future. With deeper research and further clinical trials, Tulisokibart is poised to bring more breakthroughs and advancements to the treatment landscape of ulcerative colitis.

In terms of AEs, Vedolizumab 108 mg stands out as the top‐ranked treatment choice. Vedolizumab works selectively by inhibiting the migration of gut‐homing memory T cells to the gastrointestinal tract's submucosa. The intestine‐selective mechanism of Vedolizumab may be the primary reason for its high safety profile [76]. Therefore, in the future, the benefits of many treatments should be combined to improve drug selectivity and clinical efficacy while minimizing adverse events to the greatest extent possible.

Guselkumab was deemed the best medication for increasing quality of life in our study. By inhibiting IL‐23 and IL‐17, Guselkumab effectively alleviates intestinal inflammation in patients with ulcerative colitis (UC) [77], reducing symptoms such as diarrhea, abdominal pain, and rectal bleeding, thereby enhancing their quality of life [78]. Given the chronic nature and fluctuating symptoms of UC, Guselkumab, due to its efficacy and safety, may emerge as a significant therapeutic option for UC patients, offering stable and sustained symptom control.

Current research advancements indicate that targeted therapy has demonstrated positive effects in the treatment of moderate‐to‐severe ulcerative colitis (UC). Rigorous randomized controlled trials are still required to further validate the safety and efficacy of these treatments. The application of targeted therapy continues to necessitate highly specialized care and individualized treatment strategies. To date, the pathogenesis of ulcerative colitis has not been fully elucidated. As research into the pathogenesis of UC deepens, future efforts should focus on identifying and developing drugs that target key points in the disease process, using them rationally, and minimizing adverse drug reactions, thereby providing patients with safer and more effective treatment plans.

## 5 Strength and Limitations

In comparison to previous network meta‐analyses, our study is more comprehensive in several ways. Firstly, we extracted a broader range of outcome measures, including clinical response rate, mucosal response rate, and quality of life, which were not available in previous studies. Secondly, we stratified the outcomes indicator into two treatment periods (maintenance phase and induction phase). Meanwhile, we selected current optimal medication and their dosages to conduct further analysis. This facilitated a more direct comparison of the effectiveness and safety of each top‐performing agent, which is also an innovative approach compared to previous research. By integrating relevant literature and search results, we have included as comprehensive a range of targeted therapeutic drugs [4, 7, 9, 79] as possible, while also analyzing newly developed medications. Additionally, we provide a comprehensive review of the mechanisms of action of these targeted therapeutic drugs. Consequently, our research findings are poised to offer more comprehensive and reliable evidence for drug selection in head‐to‐head studies, presenting new possibilities for improving treatment outcomes. Furthermore, it serves as a reference for treatment decision‐making in real‐world scenarios involving severe UC.

This study had several limitations. First, the patients included in this analysis were heterogeneous in key eligibility criteria and the use of concomitant medication. For instance, the NCT02039505 trial involved exclusively Japanese patients. Furthermore, the patients in ELEVATE UC 52 and ELEVATE UC 12 trials had a history of inadequate response or loss or intolerance to at least one therapy, and patients in HIBISCUS studies and the U‐ACHIEVE sub study were naive to tumor necrosis factor inhibitors. The impact of these differences may be more prominent in drugs with longer dosing intervals and half‐lives. Second, the study considered only the best drugs and dosages reported in the included literature, introducing a potential source of bias due to the different interpretations of the efficiency index across different studies, which increases the likelihood of residual confounding factors. Moreover, as described in the previous literature, any differences in the definitions of remission between trials had a low effect on relative remission versus placebo effect, and there was sufficient similarity between the two measures to allow for comparison [80]. Additionally, in the safety analysis, only the overall incidence of adverse events was considered, and specific events were not analyzed. Lastly, there is insufficient research evidence for new drugs such as Tulisokibart to treat patients with moderate‐to‐severe UC. Given the scope of our study, this may be a potential drawback. Future research addressing these limitations could provide stronger evidence for the selection of more accurate targeted therapies.

## Author Contributions

Youran Dai: Conceptualization; data curation; formal analysis; methodology; validation; writing – original draft; writing – review and editing. Wenhui Yang: Data curation; formal analysis; software; writing – original draft; writing – review and editing. Li Xu: Conceptualization; data curation; writing – original draft; writing – review and editing. Pingting Pan: Software; writing – review and editing. Shan Liu: Validation; writing – review and editing. Yingzhe Sun: Visualization; writing – review and editing. Suying Hu: Data curation; writing – review and editing. Qiushuang Li: Conceptualization; data curation; validation; writing – original draft; writing – review and editing. Fang Hu: Data curation; formal analysis; methodology; validation; writing – review and editing.

## Conflicts of Interest

The authors declare no conflicts of interest.

## Supporting information


Appendix S1.

## Data Availability

The dataset related to the conclusion is included in this article. For detailed data information, please contact the corresponding author.
