# Supplementary material for: Comparative Efficacy of Different Targeted Therapies in Patients With Moderate‐to‐Severe Ulcerative Colitis: Systematic Review/Network Meta‐Analysis and Mechanistic Overview
Source: Pharmacol Res Perspect. 2025 Jun 5;13(3):e70108. doi: 10.1002/prp2.70108 (PMC12138205; doi:10.1002/prp2.70108)
Supplement: Supplementary file 1 — Appendix S1. [file PRP2-13-e70108-s001.docx]

**Supplementary Appendix**

**Table S1. Search strategies of systematic review**

**Table S2. Risk of bias summary: review authors' judgments about each risk of bias item for each included study.**

**Table S3. Adjusted indirect treatment comparisons.**

**Table S4. SUCRA values and ranks of treatments. (i.e., clinical remission and endoscopic remission in Maintenance phase, quality of life)**

**Figure S1. Risk of bias summary.**

**Figure S2. Network of included trials in moderate to severe ulcerative colitis patients.**

**Figure S3. Efficacy outcomes (i.e., clinical remission and endoscopic remission) of targeted therapies in moderate to severe ulcerative colitis patients for the maintenance phase:** **forest plot.**

**Figure S4. Efficacy of targeted therapies in moderate to severe ulcerative colitis patients of quality of life:** **forest plot.**

**Figure S5. Funnel plots for different outcomes.**

**Table S1. Search strategies of systematic review**

| **Search strategies of PubMed** |
| --- |
| #1 ulcerative colitis [Mesh]  #2"ulcerative colitis" OR "Idiopathic Proctocolitis" OR "Colitis Gravis" OR "colitis ulcerosa" OR "colitis ulcerativa" OR "mucosal colitis" OR "ulcerative colorectitis" OR "ulcerative proctocolitis" OR "ulcerous colitis"  #3 "Ustekinumab" OR "GSK2982772" OR "AJM300" OR "Infliximab" OR "Adalimumab" OR "Golimumab" OR "Gueslkumab" OR "Basiliximab" OR "Vedolizumab" OR "Cobitolimod" OR "Eldelumab" OR "Visilizumab" OR "Daclizumab" OR "Etrolizumab" OR "PF-00547659" OR "BMS-936557" OR "Brazikumab" OR "Tofacitinib" OR "Filgotinib" OR "Upadacitinib" OR "Ozanimod" OR "Etrasimod" OR "Apremilast" OR "AJM-300" OR "Obefazimod" OR "Ivarmacitinib" OR "Izencitinib" OR "Fingolimod" OR "Mongersen" OR "Amiselimod" OR "Tulisokibart"  #6 ((#1 OR #2) AND #3 AND ((randomized controlled trial[pt] OR controlled clinical trial[pt] OR randomized[tiab] OR placebo[tiab] OR drug therapy[sh] OR randomly[tiab] OR trial[tiab] OR groups[tiab]) NOT (animals[mh] NOT humans[mh])) |
| **Search strategies of Embase** |
| #1 'ulcerative colitis'/exp  #2 ('ulcerative colitis' OR 'Idiopathic Proctocolitis' OR 'Colitis Gravis' OR 'colitis ulcerosa' OR 'colitis ulcerativa' OR 'mucosal colitis' OR 'ulcerative colorectitis' OR 'ulcerative proctocolitis' OR 'ulcerous colitis'):ti,ab,kw  #3 ('Ustekinumab' OR 'GSK2982772' OR 'AJM300' OR 'Infliximab' OR 'Adalimumab' OR 'Golimumab' OR 'Gueslkumab' OR 'Basiliximab' OR 'Vedolizumab' OR 'Cobitolimod' OR 'Eldelumab' OR 'Visilizumab' OR 'Daclizumab' OR 'Etrolizumab' OR 'PF-00547659' OR 'BMS-936557' OR 'Brazikumab' OR 'Tofacitinib' OR 'AJM-300' OR 'Filgotinib' OR 'Upadacitinib' OR 'Ozanimod' OR 'Etrasimod' OR 'Apremilast' OR 'Obefazimod' OR 'Ivarmacitinib' OR 'Izencitinib' OR 'Fingolimod' OR 'Mongersen' OR 'Amiselimod' OR 'Tulisokibart')  #6 ((#1 OR #2) AND #3 AND (('crossover procedure':de OR 'double-blind procedure':de OR 'randomized controlled trial':de OR 'single-blind procedure':de OR (random* OR factorial* OR crossover* OR cross NEXT/1 over* OR placebo* OR doubl* NEAR/1 blind* OR singl* NEAR/1 blind* OR assign* OR allocat* OR volunteer*):de,ab,ti)) |
| **Search strategies of The Cochrane Library** |
| #1 'ulcerative colitis'/exp  #2 ('ulcerative colitis' OR 'Idiopathic Proctocolitis' OR 'Colitis Gravis' OR 'colitis ulcerosa' OR 'colitis ulcerativa' OR 'mucosal colitis' OR 'ulcerative colorectitis' OR 'ulcerative proctocolitis' OR 'ulcerous colitis'):ti,ab,kw  #3 ('Ustekinumab' OR 'GSK2982772' OR 'AJM300' OR 'Infliximab' OR 'Adalimumab' OR 'Golimumab' OR 'Gueslkumab' OR 'Basiliximab' OR 'Vedolizumab' OR 'Cobitolimod' OR 'Eldelumab' OR 'Visilizumab' OR 'Daclizumab' OR 'Etrolizumab' OR 'PF-00547659' OR 'BMS-936557' OR 'Brazikumab' OR 'Tofacitinib' OR 'AJM-300' OR 'Filgotinib' OR 'Upadacitinib' OR 'Ozanimod' OR 'Etrasimod' OR 'Apremilast' OR 'Obefazimod' OR 'Ivarmacitinib' OR 'Izencitinib' OR 'Fingolimod' OR 'Mongersen' OR 'Amiselimod' OR 'Tulisokibart')  #6 ((#1 OR #2) AND #3 AND (randomized controlled trial OR controlled clinical trial OR randomized OR placebo OR drug therapy OR randomly OR trial OR groups) NOT (animals NOT humans)) |
| **Search strategies of Web of science** |
| #1TS= ("ulcerative colitis" OR "Idiopathic Proctocolitis" OR "Colitis Gravis" OR "colitis ulcerosa" OR "colitis ulcerativa" OR "mucosal colitis" OR "ulcerative colorectitis" OR "ulcerative proctocolitis" OR "ulcerous colitis")  #2 TI= ("ulcerative colitis")  #3 TS= ("Ustekinumab" OR "GSK2982772" OR "AJM300" OR "Infliximab" OR "Adalimumab" OR "Golimumab" OR "Gueslkumab" OR "Basiliximab" OR "Vedolizumab" OR "Cobitolimod" OR "Eldelumab" OR "Visilizumab" OR "Daclizumab" OR "Etrolizumab" OR "PF-00547659" OR "BMS-936557" OR "Brazikumab" OR "Tofacitinib" OR "Filgotinib" OR "Upadacitinib" OR "Ozanimod" OR "Etrasimod" OR "Apremilast" OR "AJM-300" OR "Obefazimod" OR "Ivarmacitinib" OR "Izencitinib" OR "Fingolimod" OR "Mongersen" OR "Amiselimod" OR "Tulisokibart")  #5 TS= ("randomized controlled trial" OR "controlled clinical trial" OR "randomized" OR "placebo" OR "drug therapy" OR "randomly" OR "trial" OR "groups")  #6 TS=("animals")  #7 TS=("humans")  #8 #1 AND #2  #9 #8 AND #3  #10 #5 NOT #6 NOT #7  #11 #9 AND #10 |

**Table S2. Risk of bias summary: review authors' judgments about each risk of bias item for each included study.**

| Unique ID | Randomization process | Deviations from intended interventions | Mising outcome data | Measurement of the outcome | Selection of the reported result | Overall Bias |
| --- | --- | --- | --- | --- | --- | --- |
| Raja Atreya，2020 | Low | Low | Low | Low | Low | Low |
| Walter Reinisch，2013 | Low | Low | Low | Low | Low | Low |
| William J.Sandborn,2013 | Low | Some concerns | Low | Low | Low | Some concerns |
| P. Rutgeerts，2015 | Some concerns | Low | Low | Low | Low | Some concerns |
| Silvio Danese.2022 | Low | Low | Low | Low | Low | Low |
| Bruce E. Sands.2019 | High | Some concerns | Low | Low | Low | Some concerns |
| Silvio Danese,2022 | Low | Low | Low | Low | Low | Low |
| Séverine Vermeire，2021 | High | Some concerns | Low | Low | Low | High |
| William J.Sandborn，2020 | Low | Low | Low | Low | Low | Low |
| William J. Sandborn,2014 | High | Some concerns | Low | Low | Low | Some concerns |
| William J. Sandborn,2016 | Low | Low | Low | High | Some concerns | High |
| Makoto Naganuma,2021 | Low | Low | Low | Low | Low | Low |
| Brian G Feagan,2021 | Low | Low | Low | Low | Low | Low |
| William J Sandborn,2023 | Some concerns | Low | Low | Low | Low | Some concerns |
| Severine Vermeire，2022 | Low | Low | Low | Low | Low | Low |
| David T Rubin,2022 | Low | Low | Low | Low | Low | Low |
| Lloyd Mayer,2013 | Low | Low | Some concerns | Low | Some concerns | Some concerns |
| Laurent Peyrin-Biroulet,2023 | Low | Low | Low | Low | Low | Low |
| Paul Rutgeerts,2005 | Some concerns | Some concerns | Low | Low | Low | Some concerns |
| Severine Vermeire,2022 | Low | Low | Low | Low | Some concerns | Some concerns |
| William J. Sandborn,2020 | Low | Some concerns | Low | Some concerns | Low | Some concerns |
| Bruce E. Sands 2024 | Low | Low | Low | Low | Low | Low |
| Baili Chen 2022 | Low | Low | Low | Low | Low | Low |
| William J. Sandborn 2017 | Low | Low | Low | Low | Low | Low |
| B.E. Sands, 2019 | Low | Low | Low | Some concerns | Low | Some concerns |
| G Van Assche 2006 | Low | Some concerns | Low | Some concerns | Low | Some concerns |
| Bruce E. Sands, 2024 | Low | Low | Low | Low | Low | Low |
| Séverine Vermeire 2017 | Low | Low | Low | Low | Low | Low |
| Katsuyoshi Matsuoka,2022 | Low | Low | Low | Low | Low | Low |
| Silvio Danese,2022 | Low | Low | Low | Low | Low | Low |
| Séverine Vermeire,2021 | Low | Some concerns | Low | Low | Low | Some concerns |
| Séverine Vermeire,2021 | Low | Low | Low | Low | Low | Low |
| Silvio Danese,2022 | Low | Low | Low | Low | Low | Low |
| Bruce E. Sands 2024 | Low | Low | Low | Low | Low | Low |

**Table S3. Adjusted indirect treatment comparisons.**

**A. Indirect effect estimates for induction of clinical remission.**

| ADA 160/80/40 |  |  |  |  |  |  |  |  |  |  |  |  |  |  |  |  |  |  |  |  |  |  |  |  |
| --- | --- | --- | --- | --- | --- | --- | --- | --- | --- | --- | --- | --- | --- | --- | --- | --- | --- | --- | --- | --- | --- | --- | --- | --- |
| 0.91 (0.34-2.47) | AJM 300 mg |  |  |  |  |  |  |  |  |  |  |  |  |  |  |  |  |  |  |  |  |  |  |  |
| 0.44 (0.09-2.06) | 0.49 (0.09-2.71) | Cob 250 mg |  |  |  |  |  |  |  |  |  |  |  |  |  |  |  |  |  |  |  |  |  |  |
| 2.05 (0.58-7.17) | 2.25 (0.52-9.75) | 4.63 (0.71-30.37) | Dac 2 mg/kg |  |  |  |  |  |  |  |  |  |  |  |  |  |  |  |  |  |  |  |  |  |
| 1.48 (0.45-4.92) | 1.63 (0.39-6.75) | 3.35 (0.53-21.24) | 0.72 (0.14-3.64) | Eld 10 mg/kg |  |  |  |  |  |  |  |  |  |  |  |  |  |  |  |  |  |  |  |  |
| 0.82 (0.26-2.57) | 0.90 (0.23-3.55) | 1.86 (0.30-11.31) | 0.40 (0.08-1.92) | 0.55 (0.12-2.55) | Eld 25 mg/kg |  |  |  |  |  |  |  |  |  |  |  |  |  |  |  |  |  |  |  |
| 0.30 (0.13-0.70) | 0.33 (0.10-1.03) | 0.68 (0.13-3.50) | 0.15 (0.04-0.58) | 0.20 (0.05-0.76) | 0.37 (0.10-1.30) | Etra 2 mg/kg |  |  |  |  |  |  |  |  |  |  |  |  |  |  |  |  |  |  |
| 0.81 (0.41-1.60) | 0.89 (0.32-2.48) | 1.83 (0.39-8.71) | 0.40 (0.11-1.42) | 0.55 (0.16-1.86) | 0.99 (0.31-3.16) | 2.70 (1.12-6.56) | Etro 105 mg |  |  |  |  |  |  |  |  |  |  |  |  |  |  |  |  |  |
| 0.78 (0.34-1.75) | 0.85 (0.28-2.60) | 1.75 (0.35-8.89) | 0.38 (0.10-1.46) | 0.52 (0.14-1.92) | 0.95 (0.27-3.29) | 2.59 (0.96-6.99) | 0.96 (0.41-2.24) | Fil 200 mg |  |  |  |  |  |  |  |  |  |  |  |  |  |  |  |  |
| 0.89 (0.41-1.92) | 0.98 (0.33-2.89) | 2.01 (0.41-9.96) | 0.44 (0.12-1.63) | 0.60 (0.17-2.14) | 1.09 (0.32-3.67) | 2.97 (1.15-7.71) | 1.10 (0.49-2.45) | 1.15 (0.46-2.88) | Gol 2 mg/kg |  |  |  |  |  |  |  |  |  |  |  |  |  |  |  |
| 1.05 (0.41-2.72) | 1.15 (0.34-3.90) | 2.38 (0.44-12.93) | 0.51 (0.12-2.16) | 0.71 (0.18-2.85) | 1.28 (0.34-4.89) | 3.51 (1.16-10.60) | 1.30 (0.49-3.45) | 1.35 (0.46-3.98) | 1.18 (0.42-3.34) | Gus 200 mg |  |  |  |  |  |  |  |  |  |  |  |  |  |  |
| 0.44 (0.21-0.93) | 0.48 (0.17-1.41) | 0.99 (0.20-4.87) | 0.21 (0.06-0.80) | 0.30 (0.08-1.05) | 0.54 (0.16-1.79) | 1.47 (0.57-3.75) | 0.54 (0.31-0.95) | 0.57 (0.23-1.40) | 0.49 (0.21-1.17) | 0.42 (0.15-1.17) | Inf 5 mg/kg |  |  |  |  |  |  |  |  |  |  |  |  |  |
| 0.26 (0.05-1.47) | 0.29 (0.04-1.90) | 0.59 (0.06-5.49) | 0.13 (0.02-0.98) | 0.18 (0.02-1.31) | 0.32 (0.04-2.29) | 0.87 (0.14-5.39) | 0.32 (0.06-1.85) | 0.34 (0.06-2.04) | 0.29 (0.05-1.74) | 0.25 (0.04-1.61) | 0.60 (0.10-3.50) | Iva 4 mg |  |  |  |  |  |  |  |  |  |  |  |  |
| 0.79 (0.36-1.71) | 0.87 (0.29-2.57) | 1.79 (0.36-8.85) | 0.39 (0.10-1.45) | 0.53 (0.15-1.91) | 0.96 (0.28-3.26) | 2.64 (1.01-6.86) | 0.97 (0.43-2.18) | 1.02 (0.40-2.56) | 0.89 (0.37-2.14) | 0.75 (0.26-2.14) | 1.80 (0.76-4.26) | 3.01 (0.51-17.90) | Mir 300 mg |  |  |  |  |  |  |  |  |  |  |  |
| 0.66 (0.21-2.10) | 0.73 (0.18-2.90) | 1.50 (0.24-9.21) | 0.32 (0.07-1.57) | 0.45 (0.10-2.08) | 0.81 (0.18-3.59) | 2.21 (0.61-7.99) | 0.82 (0.25-2.65) | 0.85 (0.24-3.01) | 0.74 (0.22-2.54) | 0.63 (0.16-2.44) | 1.51 (0.45-5.09) | 2.53 (0.35-18.25) | 0.84 (0.24-2.88) | Obe 50 mg |  |  |  |  |  |  |  |  |  |  |
| 0.34 (0.13-0.90) | 0.37 (0.11-1.29) | 0.77 (0.14-4.24) | 0.17 (0.04-0.71) | 0.23 (0.06-0.94) | 0.41 (0.11-1.61) | 1.13 (0.37-3.50) | 0.42 (0.15-1.14) | 0.44 (0.15-1.32) | 0.38 (0.13-1.11) | 0.32 (0.10-1.08) | 0.77 (0.27-2.21) | 1.29 (0.20-8.48) | 0.43 (0.15-1.25) | 0.51 (0.13-2.02) | Oza 0.92 mg |  |  |  |  |  |  |  |  |  |
| 1.65 (1.05-2.59) | 1.81 (0.74-4.40) | 3.73 (0.85-16.27) | 0.81 (0.25-2.59) | 1.11 (0.37-3.38) | 2.01 (0.71-5.72) | 5.50 (2.67-11.35) | 2.03 (1.22-3.39) | 2.12 (1.08-4.19) | 1.85 (1.00-3.44) | 1.57 (0.68-3.62) | 3.75 (2.07-6.80) | 6.29 (1.19-33.34) | 2.09 (1.12-3.90) | 2.49 (0.86-7.20) | 4.86 (2.04-11.57) | PBO |  |  |  |  |  |  |  |  |
| 0.22 (0.04-1.20) | 0.25 (0.04-1.56) | 0.51 (0.06-4.52) | 0.11 (0.01-0.81) | 0.15 (0.02-1.08) | 0.27 (0.04-1.88) | 0.75 (0.13-4.40) | 0.28 (0.05-1.51) | 0.29 (0.05-1.67) | 0.25 (0.04-1.42) | 0.21 (0.03-1.32) | 0.51 (0.09-2.86) | 0.86 (0.08-8.74) | 0.28 (0.05-1.61) | 0.34 (0.05-2.35) | 0.66 (0.11-4.15) | 0.14 (0.03-0.69) | PF-00547659 22.5 mg |  |  |  |  |  |  |  |
| 0.42 (0.18-0.98) | 0.47 (0.15-1.45) | 0.96 (0.19-4.91) | 0.21 (0.05-0.81) | 0.29 (0.08-1.07) | 0.52 (0.15-1.83) | 1.42 (0.52-3.88) | 0.52 (0.22-1.25) | 0.55 (0.21-1.45) | 0.48 (0.19-1.22) | 0.40 (0.14-1.20) | 0.96 (0.38-2.42) | 1.62 (0.26-9.89) | 0.54 (0.21-1.38) | 0.64 (0.18-2.29) | 1.25 (0.41-3.82) | 0.26 (0.13-0.52) | 1.89 (0.32-11.02) | Ris 1200 mg |  |  |  |  |  |  |
| 0.49 (0.20-1.18) | 0.54 (0.17-1.72) | 1.10 (0.21-5.79) | 0.24 (0.06-0.96) | 0.33 (0.09-1.26) | 0.60 (0.16-2.17) | 1.63 (0.57-4.65) | 0.60 (0.24-1.50) | 0.63 (0.23-1.74) | 0.55 (0.21-1.46) | 0.46 (0.15-1.44) | 1.11 (0.42-2.91) | 1.86 (0.30-11.64) | 0.62 (0.23-1.65) | 0.74 (0.20-2.72) | 1.44 (0.45-4.56) | 0.30 (0.14-0.63) | 2.18 (0.36-12.98) | 1.15 (0.41-3.24) | Tofa 10 mg |  |  |  |  |  |
| 0.18 (0.07-0.48) | 0.20 (0.06-0.68) | 0.40 (0.07-2.23) | 0.09 (0.02-0.37) | 0.12 (0.03-0.49) | 0.22 (0.06-0.85) | 0.59 (0.19-1.85) | 0.22 (0.08-0.60) | 0.23 (0.08-0.69) | 0.20 (0.07-0.58) | 0.17 (0.05-0.57) | 0.40 (0.14-1.16) | 0.68 (0.10-4.46) | 0.23 (0.08-0.66) | 0.27 (0.07-1.06) | 0.52 (0.15-1.80) | 0.11 (0.05-0.26) | 0.79 (0.13-4.98) | 0.42 (0.14-1.29) | 0.36 (0.11-1.16) | Tul 1000/500 mg |  |  |  |  |
| 0.16 (0.07-0.39) | 0.18 (0.06-0.57) | 0.37 (0.07-1.92) | 0.08 (0.02-0.32) | 0.11 (0.03-0.42) | 0.20 (0.06-0.72) | 0.55 (0.20-1.53) | 0.20 (0.08-0.49) | 0.21 (0.08-0.57) | 0.18 (0.07-0.48) | 0.16 (0.05-0.47) | 0.37 (0.15-0.96) | 0.63 (0.10-3.87) | 0.21 (0.08-0.54) | 0.25 (0.07-0.90) | 0.48 (0.16-1.51) | 0.10 (0.05-0.21) | 0.73 (0.12-4.32) | 0.39 (0.14-1.07) | 0.34 (0.12-0.96) | 0.92 (0.30-2.88) | Upa 45 mg |  |  |  |
| 0.83 (0.40-1.70) | 0.91 (0.32-2.60) | 1.87 (0.39-9.05) | 0.40 (0.11-1.48) | 0.56 (0.16-1.94) | 1.01 (0.31-3.31) | 2.76 (1.10-6.90) | 1.02 (0.48-2.18) | 1.07 (0.44-2.57) | 0.93 (0.40-2.14) | 0.79 (0.29-2.16) | 1.88 (0.83-4.26) | 3.16 (0.54-18.34) | 1.05 (0.45-2.43) | 1.25 (0.38-4.15) | 2.44 (0.87-6.86) | 0.50 (0.29-0.88) | 3.69 (0.67-20.42) | 1.95 (0.79-4.80) | 1.69 (0.66-4.35) | 4.65 (1.65-13.13) | 5.03 (2.00-12.64) | Uste 6 mg/kg |  |  |
| 0.52 (0.12-2.23) | 0.58 (0.11-2.96) | 1.19 (0.16-8.90) | 0.26 (0.04-1.56) | 0.35 (0.06-2.07) | 0.64 (0.11-3.60) | 1.75 (0.37-8.28) | 0.65 (0.15-2.81) | 0.68 (0.15-3.13) | 0.59 (0.13-2.66) | 0.50 (0.10-2.50) | 1.19 (0.27-5.34) | 2.00 (0.23-17.39) | 0.66 (0.15-3.01) | 0.79 (0.14-4.50) | 1.55 (0.30-7.86) | 0.32 (0.08-1.26) | 2.34 (0.28-19.53) | 1.24 (0.26-5.80) | 1.07 (0.22-5.16) | 2.95 (0.58-15.03) | 3.19 (0.67-15.14) | 0.63 (0.14-2.80) | Ved 108 mg |  |
| 0.70 (0.43-1.13) | 0.76 (0.27-2.13) | 1.58 (0.33-7.49) | 0.34 (0.10-1.22) | 0.47 (0.14-1.60) | 0.85 (0.26-2.72) | 2.33 (0.96-5.64) | 0.86 (0.42-1.77) | 0.90 (0.38-2.10) | 0.78 (0.35-1.75) | 0.66 (0.25-1.77) | 1.58 (0.72-3.47) | 2.66 (0.46-15.21) | 0.88 (0.39-1.98) | 1.05 (0.32-3.42) | 2.05 (0.75-5.62) | 0.42 (0.25-0.70) | 3.11 (0.57-16.93) | 1.64 (0.69-3.92) | 1.43 (0.57-3.56) | 3.92 (1.42-10.77) | 4.24 (1.74-10.33) | 0.84 (0.39-1.80) | 1.33 (0.31-5.75) | Ved 300 mg |

**B. Indirect effect estimates for induction of clinical response.**

| ADA 160/80/40 |  |  |  |  |  |  |  |  |  |  |  |  |  |  |  |  | . | . | . | . | . | . |
| --- | --- | --- | --- | --- | --- | --- | --- | --- | --- | --- | --- | --- | --- | --- | --- | --- | --- | --- | --- | --- | --- | --- |
| 0.53 (0.16-1.75) | AJM 300 960 mg |  |  |  |  |  |  |  |  |  |  |  |  |  |  |  |  |  |  |  |  |  |
| 2.50 (0.66-9.51) | 4.69 (1.00-22.07) | Cob 250 mg |  |  |  |  |  |  |  |  |  |  |  |  |  |  |  |  |  |  |  |  |
| 2.59 (0.67-10.04) | 4.87 (1.02-23.24) | 1.04 (0.19-5.55) | Dac 2 mg/kg |  |  |  |  |  |  |  |  |  |  |  |  |  |  |  |  |  |  |  |
| 0.75 (0.21-2.68) | 1.40 (0.31-6.28) | 0.30 (0.06-1.51) | 0.29 (0.06-1.47) | Eld 10 mg/kg |  |  |  |  |  |  |  |  |  |  |  |  |  |  |  |  |  |  |
| 0.85 (0.26-2.83) | 1.61 (0.38-6.70) | 0.34 (0.07-1.62) | 0.33 (0.07-1.58) | 1.14 (0.25-5.15) | Eld 25 mg/kg |  |  |  |  |  |  |  |  |  |  |  |  |  |  |  |  |  |
| 0.56 (0.21-1.47) | 1.05 (0.30-3.65) | 0.22 (0.06-0.89) | 0.22 (0.05-0.88) | 0.75 (0.20-2.83) | 0.65 (0.19-2.28) | Etra 2 mg/kg |  |  |  |  |  |  |  |  |  |  |  |  |  |  |  |  |
| 0.47 (0.11-2.06) | 0.88 (0.16-4.69) | 0.19 (0.03-1.11) | 0.18 (0.03-1.09) | 0.62 (0.11-3.56) | 0.55 (0.10-2.93) | 0.83 (0.18-3.84) | Etro 105 mg |  |  |  |  |  |  |  |  |  |  |  |  |  |  |  |
| 0.83 (0.32-2.12) | 1.55 (0.46-5.27) | 0.33 (0.08-1.29) | 0.32 (0.08-1.27) | 1.11 (0.30-4.09) | 0.97 (0.28-3.30) | 1.48 (0.54-4.04) | 1.77 (0.39-8.01) | Gol 2 mg/kg |  |  |  |  |  |  |  |  |  |  |  |  |  |  |
| 0.40 (0.12-1.29) | 0.75 (0.18-3.08) | 0.16 (0.03-0.74) | 0.15 (0.03-0.73) | 0.54 (0.12-2.36) | 0.47 (0.11-1.93) | 0.71 (0.21-2.44) | 0.86 (0.16-4.53) | 0.48 (0.14-1.61) | Gus 200 mg |  |  |  |  |  |  |  |  |  |  |  |  |  |
| 0.43 (0.14-1.37) | 0.82 (0.20-3.28) | 0.17 (0.04-0.79) | 0.17 (0.04-0.78) | 0.58 (0.13-2.52) | 0.51 (0.13-2.05) | 0.78 (0.23-2.59) | 0.93 (0.36-2.38) | 0.53 (0.16-1.71) | 1.09 (0.27-4.30) | Inf 5 mg/kg |  |  |  |  |  |  |  |  |  |  |  |  |
| 0.76 (0.19-3.04) | 1.43 (0.29-7.01) | 0.30 (0.06-1.67) | 0.29 (0.05-1.63) | 1.02 (0.19-5.35) | 0.89 (0.18-4.39) | 1.36 (0.32-5.70) | 1.63 (0.26-10.07) | 0.92 (0.22-3.78) | 1.90 (0.39-9.22) | 1.75 (0.37-8.33) | Iva 4 mg |  |  |  |  |  |  |  |  |  |  |  |
| 0.70 (0.24-2.00) | 1.31 (0.35-4.87) | 0.28 (0.07-1.19) | 0.27 (0.06-1.16) | 0.94 (0.23-3.76) | 0.82 (0.22-3.05) | 1.25 (0.41-3.80) | 1.50 (0.31-7.29) | 0.85 (0.29-2.51) | 1.75 (0.48-6.39) | 1.61 (0.45-5.75) | 0.92 (0.21-4.10) | Mir 300 mg |  |  |  |  |  |  |  |  |  |  |
| 0.49 (0.16-1.49) | 0.93 (0.24-3.58) | 0.20 (0.05-0.87) | 0.19 (0.04-0.85) | 0.66 (0.16-2.76) | 0.58 (0.15-2.24) | 0.88 (0.28-2.81) | 1.06 (0.21-5.32) | 0.60 (0.19-1.86) | 1.24 (0.33-4.70) | 1.14 (0.31-4.23) | 0.65 (0.14-3.00) | 0.71 (0.21-2.42) | Oza 0.92 mg |  |  |  |  |  |  |  |  |  |
| 1.67 (0.88-3.15) | 3.13 (1.14-8.57) | 0.67 (0.21-2.16) | 0.64 (0.19-2.13) | 2.23 (0.74-6.77) | 1.95 (0.71-5.37) | 2.98 (1.43-6.18) | 3.57 (0.93-13.65) | 2.01 (1.01-4.02) | 4.17 (1.55-11.18) | 3.83 (1.47-9.99) | 2.20 (0.64-7.55) | 2.38 (1.03-5.51) | 3.37 (1.37-8.25) | PBO |  |  |  |  |  |  |  |  |
| 0.53 (0.16-1.83) | 1.00 (0.23-4.32) | 0.21 (0.04-1.04) | 0.21 (0.04-1.02) | 0.72 (0.15-3.31) | 0.63 (0.14-2.70) | 0.96 (0.27-3.45) | 1.15 (0.21-6.31) | 0.65 (0.18-2.28) | 1.34 (0.32-5.67) | 1.23 (0.30-5.11) | 0.70 (0.14-3.57) | 0.76 (0.20-2.94) | 1.08 (0.27-4.31) | 0.32 (0.11-0.92) | PF-00547659 22.5 mg |  |  |  |  |  |  |  |
| 0.51 (0.18-1.47) | 0.96 (0.26-3.58) | 0.21 (0.05-0.87) | 0.20 (0.05-0.85) | 0.69 (0.17-2.77) | 0.60 (0.16-2.24) | 0.92 (0.30-2.80) | 1.10 (0.23-5.36) | 0.62 (0.21-1.84) | 1.28 (0.35-4.70) | 1.18 (0.33-4.23) | 0.68 (0.15-3.02) | 0.73 (0.22-2.41) | 1.04 (0.30-3.55) | 0.31 (0.13-0.71) | 0.96 (0.25-3.70) | Ris 1200 mg |  |  |  |  |  |  |
| 0.55 (0.19-1.58) | 1.03 (0.27-3.83) | 0.22 (0.05-0.93) | 0.21 (0.05-0.91) | 0.73 (0.18-2.96) | 0.64 (0.17-2.40) | 0.98 (0.32-3.00) | 1.17 (0.24-5.73) | 0.66 (0.22-1.98) | 1.36 (0.37-5.03) | 1.26 (0.35-4.52) | 0.72 (0.16-3.22) | 0.78 (0.24-2.58) | 1.10 (0.32-3.80) | 0.33 (0.14-0.77) | 1.02 (0.26-3.96) | 1.06 (0.32-3.52) | Tofa 10 mg |  |  |  |  |  |
| 0.69 (0.21-2.20) | 1.29 (0.32-5.24) | 0.27 (0.06-1.27) | 0.26 (0.06-1.24) | 0.92 (0.21-4.03) | 0.80 (0.20-3.28) | 1.23 (0.36-4.15) | 1.47 (0.28-7.72) | 0.83 (0.25-2.74) | 1.72 (0.43-6.88) | 1.58 (0.40-6.20) | 0.90 (0.19-4.36) | 0.98 (0.27-3.55) | 1.39 (0.37-5.21) | 0.41 (0.16-1.09) | 1.28 (0.31-5.39) | 1.34 (0.37-4.85) | 1.26 (0.34-4.59) | Tul 1000/500 mg |  |  |  |  |
| 0.21 (0.08-0.55) | 0.39 (0.11-1.35) | 0.08 (0.02-0.33) | 0.08 (0.02-0.33) | 0.28 (0.07-1.05) | 0.24 (0.07-0.85) | 0.37 (0.13-1.04) | 0.45 (0.10-2.05) | 0.25 (0.09-0.68) | 0.52 (0.15-1.77) | 0.48 (0.15-1.59) | 0.28 (0.07-1.15) | 0.30 (0.10-0.90) | 0.42 (0.13-1.33) | 0.13 (0.06-0.26) | 0.39 (0.11-1.40) | 0.41 (0.14-1.23) | 0.38 (0.13-1.17) | 0.30 (0.09-1.02) | Upa 45 mg |  |  |  |
| 0.75 (0.31-1.81) | 1.40 (0.43-4.56) | 0.30 (0.08-1.12) | 0.29 (0.08-1.10) | 1.00 (0.28-3.55) | 0.87 (0.27-2.85) | 1.34 (0.52-3.46) | 1.60 (0.37-6.99) | 0.90 (0.36-2.27) | 1.87 (0.58-5.97) | 1.72 (0.55-5.35) | 0.98 (0.25-3.91) | 1.07 (0.38-3.02) | 1.51 (0.51-4.47) | 0.45 (0.24-0.83) | 1.40 (0.41-4.72) | 1.46 (0.51-4.12) | 1.37 (0.48-3.91) | 1.09 (0.34-3.44) | 3.57 (1.39-9.15) | Uste 6 mg/kg |  |  |
| 0.37 (0.11-1.28) | 0.70 (0.16-3.02) | 0.15 (0.03-0.73) | 0.14 (0.03-0.71) | 0.50 (0.11-2.31) | 0.44 (0.10-1.89) | 0.67 (0.18-2.41) | 0.80 (0.14-4.41) | 0.45 (0.13-1.60) | 0.93 (0.22-3.97) | 0.86 (0.21-3.58) | 0.49 (0.10-2.50) | 0.53 (0.14-2.06) | 0.75 (0.19-3.02) | 0.22 (0.08-0.65) | 0.70 (0.16-3.11) | 0.73 (0.19-2.81) | 0.68 (0.18-2.66) | 0.54 (0.13-2.29) | 1.78 (0.49-6.39) | 0.50 (0.15-1.70) | Ved 108 mg |  |
| 0.97 (0.38-2.46) | 1.82 (0.54-6.14) | 0.39 (0.10-1.51) | 0.37 (0.09-1.48) | 1.30 (0.35-4.77) | 1.14 (0.34-3.85) | 1.74 (0.64-4.70) | 2.08 (0.46-9.35) | 1.17 (0.45-3.09) | 2.43 (0.73-8.04) | 2.23 (0.69-7.22) | 1.28 (0.31-5.24) | 1.39 (0.47-4.09) | 1.96 (0.64-6.04) | 0.58 (0.30-1.15) | 1.82 (0.52-6.36) | 1.89 (0.64-5.58) | 1.78 (0.60-5.29) | 1.41 (0.43-4.64) | 4.64 (1.73-12.43) | 1.30 (0.52-3.24) | 2.61 (0.74-9.18) | Ved 300 mg |

**C. Indirect effect estimates for induction of endoscopic response.**

| AJM 300 mg |  |  |  |  |  |  |  |  |  |  |  |  |  |
| --- | --- | --- | --- | --- | --- | --- | --- | --- | --- | --- | --- | --- | --- |
| 0.15 (0.02-0.96) | Cob 250 mg |  |  |  |  |  |  |  |  |  |  |  |  |
| 8.45 (1.85-38.61) | 56.33 (11.62-273.08) | Dac 2 mg/kg |  |  |  |  |  |  |  |  |  |  |  |
| 1.45 (0.37-5.69) | 9.66 (2.30-40.49) | 0.17 (0.07-0.44) | Etra 2 mg/kg |  |  |  |  |  |  |  |  |  |  |
| 1.66 (0.38-7.19) | 11.06 (2.40-50.99) | 0.20 (0.07-0.58) | 1.15 (0.48-2.73) | Gus 200 mg |  |  |  |  |  |  |  |  |  |
| 1.92 (0.23-16.13) | 12.80 (1.46-112.22) | 0.23 (0.03-1.50) | 1.33 (0.23-7.77) | 1.16 (0.18-7.34) | Iva 4 mg |  |  |  |  |  |  |  |  |
| 2.44 (0.65-9.10) | 16.26 (4.07-64.94) | 0.29 (0.12-0.69) | 1.68 (0.95-3.00) | 1.47 (0.67-3.22) | 1.27 (0.23-7.17) | Mir 300 mg |  |  |  |  |  |  |  |
| 1.36 (0.34-5.47) | 9.09 (2.13-38.89) | 0.16 (0.06-0.43) | 0.94 (0.45-1.95) | 0.82 (0.33-2.02) | 0.71 (0.12-4.23) | 0.56 (0.30-1.04) | Oza 0.92 mg |  |  |  |  |  |  |
| 5.20 (1.45-18.69) | 34.67 (9.00-133.56) | 0.62 (0.27-1.40) | 3.59 (2.21-5.83) | 3.13 (1.53-6.43) | 2.71 (0.49-14.84) | 2.13 (1.56-2.91) | 3.81 (2.22-6.55) | PBO |  |  |  |  |  |
| 1.53 (0.36-6.44) | 10.23 (2.29-45.72) | 0.18 (0.06-0.52) | 1.06 (0.47-2.38) | 0.92 (0.35-2.44) | 0.80 (0.13-4.94) | 0.63 (0.31-1.29) | 1.12 (0.48-2.62) | 0.29 (0.15-0.57) | Ris 1200 mg |  |  |  |  |
| 1.23 (0.24-6.32) | 8.20 (1.51-44.51) | 0.15 (0.04-0.54) | 0.85 (0.27-2.63) | 0.74 (0.21-2.58) | 0.64 (0.09-4.66) | 0.50 (0.17-1.47) | 0.90 (0.28-2.87) | 0.24 (0.09-0.66) | 0.80 (0.24-2.69) | Tofa 10 mg |  |  |  |
| 2.17 (0.52-9.05) | 14.45 (3.25-64.25) | 0.26 (0.09-0.72) | 1.50 (0.67-3.34) | 1.31 (0.50-3.41) | 1.13 (0.18-6.95) | 0.89 (0.44-1.81) | 1.59 (0.69-3.67) | 0.42 (0.22-0.79) | 1.41 (0.57-3.52) | 1.76 (0.53-5.88) | Tul 1000/500 mg |  |  |
| 0.61 (0.16-2.34) | 4.05 (0.98-16.67) | 0.07 (0.03-0.18) | 0.42 (0.22-0.80) | 0.37 (0.16-0.84) | 0.32 (0.05-1.83) | 0.25 (0.15-0.42) | 0.45 (0.22-0.89) | 0.12 (0.08-0.18) | 0.40 (0.18-0.86) | 0.49 (0.16-1.50) | 0.28 (0.13-0.60) | Upa 45 mg |  |
| 1.09 (0.25-4.78) | 7.25 (1.55-33.84) | 0.13 (0.04-0.39) | 0.75 (0.31-1.83) | 0.66 (0.23-1.84) | 0.57 (0.09-3.63) | 0.45 (0.20-1.00) | 0.80 (0.32-2.00) | 0.21 (0.10-0.44) | 0.71 (0.26-1.91) | 0.88 (0.25-3.13) | 0.50 (0.19-1.34) | 1.79 (0.76-4.22) | Ved 108 mg |

**D. Indirect effect estimates for mucosal healing.**

| ADA 160/80/40 |  |  |  |  |  |  |  |  |  |  |  |  |  |  |  |
| --- | --- | --- | --- | --- | --- | --- | --- | --- | --- | --- | --- | --- | --- | --- | --- |
| 0.87 (0.31-2.45) | Eld 10 mg/kg |  |  |  |  |  |  |  |  |  |  |  |  |  |  |
| 1.03 (0.39-2.67) | 1.18 (0.38-3.67) | Eld 25 mg/kg |  |  |  |  |  |  |  |  |  |  |  |  |  |
| 0.91 (0.43-1.93) | 1.05 (0.40-2.75) | 0.88 (0.37-2.12) | Gol 2 mg/kg |  |  |  |  |  |  |  |  |  |  |  |  |
| 0.44 (0.15-1.31) | 0.51 (0.15-1.77) | 0.43 (0.13-1.40) | 0.49 (0.18-1.35) | Gus 200 mg |  |  |  |  |  |  |  |  |  |  |  |
| 0.39 (0.16-0.93) | 0.45 (0.16-1.30) | 0.38 (0.14-1.01) | 0.43 (0.20-0.94) | 0.88 (0.29-2.64) | Inf 5 mg/kg |  |  |  |  |  |  |  |  |  |  |
| 0.53 (0.24-1.15) | 0.61 (0.23-1.63) | 0.52 (0.21-1.26) | 0.58 (0.30-1.15) | 1.19 (0.42-3.33) | 1.35 (0.61-3.01) | Mir 300 mg |  |  |  |  |  |  |  |  |  |
| 0.31 (0.10-0.92) | 0.35 (0.10-1.24) | 0.30 (0.09-0.98) | 0.34 (0.12-0.95) | 0.69 (0.19-2.51) | 0.78 (0.26-2.39) | 0.58 (0.20-1.66) | Oza 0.92 mg |  |  |  |  |  |  |  |  |
| 1.25 (0.69-2.26) | 1.44 (0.61-3.36) | 1.21 (0.58-2.56) | 1.37 (0.87-2.18) | 2.80 (1.14-6.90) | 3.18 (1.70-5.95) | 2.36 (1.43-3.88) | 4.05 (1.62-10.17) | PBO |  |  |  |  |  |  |  |
| 0.28 (0.08-0.93) | 0.32 (0.08-1.23) | 0.27 (0.08-0.98) | 0.31 (0.10-0.96) | 0.63 (0.16-2.48) | 0.71 (0.21-2.40) | 0.53 (0.17-1.67) | 0.91 (0.23-3.64) | 0.22 (0.08-0.63) | PF-00547659 22.5 mg |  |  |  |  |  |  |
| 0.32 (0.14-0.73) | 0.37 (0.13-1.02) | 0.31 (0.12-0.79) | 0.35 (0.17-0.73) | 0.72 (0.25-2.08) | 0.82 (0.35-1.90) | 0.61 (0.29-1.29) | 1.04 (0.36-3.06) | 0.26 (0.15-0.45) | 1.15 (0.35-3.75) | Ris 1200 mg |  |  |  |  |  |
| 0.46 (0.21-1.02) | 0.53 (0.20-1.45) | 0.45 (0.18-1.12) | 0.51 (0.25-1.02) | 1.04 (0.37-2.95) | 1.18 (0.52-2.67) | 0.87 (0.42-1.80) | 1.50 (0.52-4.33) | 0.37 (0.22-0.63) | 1.66 (0.52-5.31) | 1.44 (0.67-3.10) | Tofa 10 mg |  |  |  |  |
| 0.42 (0.16-1.08) | 0.48 (0.16-1.49) | 0.41 (0.14-1.16) | 0.46 (0.19-1.10) | 0.94 (0.29-3.01) | 1.07 (0.41-2.81) | 0.79 (0.33-1.93) | 1.36 (0.42-4.42) | 0.34 (0.16-0.70) | 1.50 (0.42-5.36) | 1.30 (0.52-3.29) | 0.91 (0.37-2.24) | Tul 1000/500 mg |  |  |  |
| 0.14 (0.04-0.44) | 0.16 (0.04-0.59) | 0.14 (0.04-0.46) | 0.15 (0.05-0.45) | 0.31 (0.08-1.19) | 0.36 (0.11-1.14) | 0.26 (0.09-0.79) | 0.45 (0.12-1.74) | 0.11 (0.04-0.30) | 0.50 (0.12-2.09) | 0.44 (0.14-1.34) | 0.30 (0.10-0.92) | 0.33 (0.10-1.13) | Upa 45 mg |  |  |
| 0.53 (0.23-1.24) | 0.62 (0.22-1.73) | 0.52 (0.20-1.35) | 0.59 (0.28-1.24) | 1.20 (0.41-3.52) | 1.36 (0.58-3.22) | 1.01 (0.47-2.19) | 1.74 (0.58-5.18) | 0.43 (0.24-0.77) | 1.92 (0.58-6.33) | 1.67 (0.74-3.76) | 1.16 (0.53-2.54) | 1.28 (0.50-3.28) | 3.82 (1.22-11.93) | Uste 6 mg/kg |  |
| 0.74 (0.35-1.55) | 0.85 (0.32-2.22) | 0.72 (0.30-1.71) | 0.81 (0.43-1.54) | 1.65 (0.60-4.53) | 1.88 (0.87-4.06) | 1.39 (0.71-2.72) | 2.39 (0.86-6.66) | 0.59 (0.38-0.92) | 2.64 (0.85-8.19) | 2.29 (1.12-4.70) | 1.59 (0.80-3.18) | 1.76 (0.74-4.16) | 5.26 (1.80-15.39) | 1.38 (0.66-2.88) | Ved 300 mg |

**E. Indirect effect estimates for AEs.**

| ADA 160/80/40 |  |  |  |  |  |  |  |  |  |  |  |  |  |  |  |  |  |  |  |  |  |  |  |  |
| --- | --- | --- | --- | --- | --- | --- | --- | --- | --- | --- | --- | --- | --- | --- | --- | --- | --- | --- | --- | --- | --- | --- | --- | --- |
| 1.07 (0.43-2.64) | AJM 300 mg | . |  |  |  |  |  |  |  |  |  |  |  |  |  |  |  |  |  |  |  |  |  |  |
| 1.73 (0.57-5.28) | 1.62 (0.44-5.90) | Cob 250 mg |  |  |  |  |  |  |  |  |  |  |  |  |  |  |  |  |  |  |  |  |  |  |
| 0.96 (0.31-3.04) | 0.90 (0.24-3.39) | 0.56 (0.13-2.44) | Dac 2 mg/kg |  |  |  |  |  |  |  |  |  |  |  |  |  |  |  |  |  |  |  |  |  |
| 0.72 (0.25-2.09) | 0.68 (0.20-2.35) | 0.42 (0.10-1.71) | 0.75 (0.18-3.15) | Eld 10 mg/kg |  |  |  |  |  |  |  |  |  |  |  |  |  |  |  |  |  |  |  |  |
| 0.96 (0.37-2.52) | 0.90 (0.28-2.89) | 0.56 (0.15-2.12) | 1.00 (0.25-3.90) | 1.33 (0.37-4.81) | Eld 25 mg/kg |  |  |  |  |  |  |  |  |  |  |  |  |  |  |  |  |  |  |  |
| 0.71 (0.34-1.48) | 0.67 (0.25-1.78) | 0.41 (0.13-1.34) | 0.74 (0.22-2.47) | 0.98 (0.32-3.02) | 0.74 (0.26-2.09) | Etra 2 mg/kg |  |  |  |  |  |  |  |  |  |  |  |  |  |  |  |  |  |  |
| 1.28 (0.67-2.46) | 1.20 (0.48-3.02) | 0.74 (0.24-2.30) | 1.33 (0.42-4.26) | 1.77 (0.60-5.18) | 1.34 (0.50-3.57) | 1.80 (0.85-3.83) | Etro 105 mg |  |  |  |  |  |  |  |  |  |  |  |  |  |  |  |  |  |
| 1.17 (0.54-2.51) | 1.09 (0.40-3.00) | 0.68 (0.20-2.25) | 1.21 (0.35-4.15) | 1.61 (0.51-5.08) | 1.22 (0.42-3.52) | 1.64 (0.70-3.86) | 0.91 (0.41-2.00) | Fil 200 mg |  |  |  |  |  |  |  |  |  |  |  |  |  |  |  |  |
| 0.88 (0.43-1.80) | 0.82 (0.31-2.17) | 0.51 (0.16-1.64) | 0.91 (0.27-3.04) | 1.21 (0.40-3.70) | 0.92 (0.33-2.56) | 1.24 (0.55-2.78) | 0.69 (0.33-1.44) | 0.75 (0.32-1.75) | Gol 2 mg/kg |  |  |  |  |  |  |  |  |  |  |  |  |  |  |  |
| 1.68 (0.69-4.11) | 1.57 (0.52-4.76) | 0.97 (0.27-3.52) | 1.74 (0.47-6.49) | 2.32 (0.67-7.98) | 1.75 (0.55-5.57) | 2.36 (0.89-6.24) | 1.31 (0.52-3.26) | 1.44 (0.53-3.90) | 1.91 (0.73-5.00) | Gus 200 mg |  |  |  |  |  |  |  |  |  |  |  |  |  |  |
| 1.14 (0.52-2.49) | 1.07 (0.39-2.95) | 0.66 (0.20-2.21) | 1.18 (0.34-4.09) | 1.57 (0.50-5.00) | 1.19 (0.41-3.47) | 1.60 (0.68-3.81) | 0.89 (0.49-1.62) | 0.98 (0.40-2.39) | 1.30 (0.55-3.05) | 0.68 (0.25-1.86) | Inf 5 mg/kg |  |  |  |  |  |  |  |  |  |  |  |  |  |
| 0.71 (0.23-2.18) | 0.66 (0.18-2.43) | 0.41 (0.10-1.76) | 0.73 (0.17-3.23) | 0.98 (0.24-4.01) | 0.74 (0.19-2.82) | 0.99 (0.30-3.26) | 0.55 (0.18-1.72) | 0.60 (0.18-2.03) | 0.80 (0.25-2.62) | 0.42 (0.12-1.54) | 0.62 (0.18-2.09) | Iva 4 mg |  |  |  |  |  |  |  |  |  |  |  |  |
| 1.12 (0.53-2.38) | 1.05 (0.39-2.85) | 0.65 (0.20-2.14) | 1.17 (0.34-3.96) | 1.55 (0.50-4.83) | 1.17 (0.41-3.35) | 1.58 (0.68-3.66) | 0.88 (0.40-1.90) | 0.96 (0.40-2.30) | 1.28 (0.56-2.93) | 0.67 (0.25-1.80) | 0.98 (0.41-2.38) | 1.59 (0.48-5.28) | Mir 300 mg |  |  |  |  |  |  |  |  |  |  |  |
| 0.79 (0.29-2.13) | 0.74 (0.22-2.43) | 0.46 (0.12-1.78) | 0.82 (0.20-3.27) | 1.09 (0.29-4.04) | 0.82 (0.24-2.83) | 1.11 (0.38-3.22) | 0.61 (0.22-1.69) | 0.67 (0.23-2.00) | 0.90 (0.31-2.58) | 0.47 (0.14-1.53) | 0.69 (0.23-2.07) | 1.11 (0.28-4.38) | 0.70 (0.24-2.06) | Obe 50 mg |  |  |  |  |  |  |  |  |  |  |
| 1.06 (0.47-2.40) | 1.00 (0.35-2.83) | 0.62 (0.18-2.11) | 1.10 (0.31-3.90) | 1.47 (0.45-4.77) | 1.11 (0.37-3.32) | 1.50 (0.61-3.67) | 0.83 (0.36-1.91) | 0.91 (0.36-2.29) | 1.21 (0.50-2.94) | 0.63 (0.23-1.78) | 0.93 (0.37-2.38) | 1.51 (0.44-5.20) | 0.95 (0.38-2.36) | 1.35 (0.44-4.16) | Oza 0.92 mg |  |  |  |  |  |  |  |  |  |
| 1.05 (0.68-1.64) | 0.98 (0.45-2.17) | 0.61 (0.22-1.70) | 1.09 (0.38-3.15) | 1.45 (0.55-3.80) | 1.09 (0.46-2.58) | 1.48 (0.83-2.65) | 0.82 (0.51-1.32) | 0.90 (0.48-1.68) | 1.20 (0.68-2.11) | 0.63 (0.29-1.37) | 0.92 (0.49-1.75) | 1.49 (0.53-4.19) | 0.94 (0.51-1.72) | 1.34 (0.55-3.26) | 0.99 (0.50-1.95) | PBO |  |  |  |  |  |  |  |  |
| 1.14 (0.43-2.99) | 1.07 (0.33-3.42) | 0.66 (0.17-2.51) | 1.18 (0.30-4.62) | 1.57 (0.43-5.70) | 1.19 (0.35-3.99) | 1.60 (0.57-4.51) | 0.89 (0.33-2.37) | 0.97 (0.34-2.81) | 1.30 (0.46-3.62) | 0.68 (0.21-2.16) | 1.00 (0.34-2.91) | 1.61 (0.42-6.18) | 1.01 (0.35-2.90) | 1.45 (0.42-4.99) | 1.07 (0.36-3.20) | 1.08 (0.46-2.55) | PF-00547659 22.5 mg |  |  |  |  |  |  |  |
| 1.43 (0.67-3.04) | 1.34 (0.49-3.63) | 0.83 (0.25-2.73) | 1.48 (0.44-5.05) | 1.97 (0.63-6.17) | 1.49 (0.52-4.27) | 2.01 (0.86-4.68) | 1.11 (0.51-2.42) | 1.22 (0.51-2.93) | 1.63 (0.71-3.74) | 0.85 (0.32-2.29) | 1.25 (0.52-3.04) | 2.02 (0.61-6.74) | 1.27 (0.54-3.01) | 1.82 (0.61-5.36) | 1.34 (0.54-3.36) | 1.36 (0.74-2.51) | 1.25 (0.44-3.60) | Ris 1200 mg |  |  |  |  |  |  |
| 1.10 (0.51-2.36) | 1.03 (0.38-2.81) | 0.63 (0.19-2.11) | 1.14 (0.33-3.90) | 1.51 (0.48-4.76) | 1.14 (0.40-3.30) | 1.54 (0.66-3.62) | 0.86 (0.39-1.88) | 0.94 (0.39-2.27) | 1.25 (0.54-2.90) | 0.65 (0.24-1.77) | 0.96 (0.39-2.35) | 1.55 (0.46-5.20) | 0.98 (0.41-2.33) | 1.39 (0.47-4.14) | 1.03 (0.41-2.59) | 1.04 (0.56-1.95) | 0.96 (0.33-2.78) | 0.77 (0.32-1.84) | Tofa 10 mg |  |  |  |  |  |
| 0.96 (0.38-2.40) | 0.89 (0.29-2.77) | 0.55 (0.15-2.04) | 0.99 (0.26-3.76) | 1.32 (0.38-4.63) | 0.99 (0.31-3.23) | 1.34 (0.50-3.64) | 0.74 (0.29-1.91) | 0.82 (0.29-2.27) | 1.09 (0.40-2.92) | 0.57 (0.19-1.75) | 0.84 (0.30-2.35) | 1.35 (0.36-5.03) | 0.85 (0.31-2.34) | 1.21 (0.36-4.05) | 0.90 (0.31-2.58) | 0.91 (0.40-2.04) | 0.84 (0.26-2.72) | 0.67 (0.24-1.84) | 0.87 (0.31-2.42) | Tul 1000/500 mg |  |  |  |  |
| 1.04 (0.52-2.06) | 0.97 (0.38-2.51) | 0.60 (0.19-1.90) | 1.08 (0.33-3.52) | 1.44 (0.48-4.29) | 1.08 (0.40-2.96) | 1.46 (0.67-3.20) | 0.81 (0.40-1.65) | 0.89 (0.39-2.01) | 1.18 (0.55-2.56) | 0.62 (0.24-1.58) | 0.91 (0.40-2.08) | 1.47 (0.46-4.70) | 0.93 (0.42-2.06) | 1.32 (0.47-3.72) | 0.98 (0.41-2.31) | 0.99 (0.59-1.67) | 0.91 (0.33-2.49) | 0.73 (0.33-1.63) | 0.95 (0.42-2.14) | 1.09 (0.42-2.85) | Upa 45 mg |  |  |  |
| 0.95 (0.51-1.79) | 0.89 (0.36-2.21) | 0.55 (0.18-1.69) | 0.99 (0.31-3.12) | 1.31 (0.45-3.79) | 0.99 (0.38-2.61) | 1.34 (0.64-2.79) | 0.74 (0.38-1.43) | 0.81 (0.38-1.75) | 1.08 (0.53-2.23) | 0.57 (0.23-1.39) | 0.83 (0.38-1.82) | 1.35 (0.44-4.16) | 0.85 (0.40-1.80) | 1.21 (0.44-3.28) | 0.89 (0.40-2.02) | 0.90 (0.58-1.41) | 0.84 (0.32-2.20) | 0.67 (0.31-1.42) | 0.87 (0.40-1.87) | 1.00 (0.40-2.51) | 0.91 (0.46-1.82) | Uste 6 mg/kg |  |  |
| 1.87 (0.67-5.18) | 1.75 (0.52-5.87) | 1.08 (0.27-4.28) | 1.93 (0.47-7.89) | 2.57 (0.68-9.75) | 1.94 (0.55-6.84) | 2.62 (0.88-7.79) | 1.45 (0.51-4.10) | 1.60 (0.52-4.85) | 2.12 (0.72-6.25) | 1.11 (0.33-3.71) | 1.63 (0.53-5.02) | 2.64 (0.66-10.55) | 1.66 (0.55-5.00) | 2.37 (0.66-8.55) | 1.75 (0.56-5.51) | 1.77 (0.71-4.46) | 1.64 (0.47-5.76) | 1.31 (0.43-3.95) | 1.70 (0.56-5.17) | 1.95 (0.57-6.65) | 1.79 (0.62-5.17) | 1.96 (0.70-5.46) | Ved 108 mg |  |
| 1.03 (0.64-1.66) | 0.96 (0.39-2.37) | 0.59 (0.19-1.81) | 1.07 (0.34-3.36) | 1.42 (0.49-4.08) | 1.07 (0.41-2.81) | 1.44 (0.70-3.00) | 0.80 (0.42-1.53) | 0.88 (0.41-1.89) | 1.17 (0.57-2.39) | 0.61 (0.25-1.50) | 0.90 (0.41-1.96) | 1.45 (0.47-4.48) | 0.91 (0.43-1.94) | 1.30 (0.48-3.53) | 0.96 (0.43-2.17) | 0.98 (0.63-1.52) | 0.90 (0.34-2.36) | 0.72 (0.34-1.53) | 0.94 (0.44-2.01) | 1.08 (0.43-2.70) | 0.99 (0.50-1.95) | 1.08 (0.58-2.02) | 0.55 (0.20-1.53) | Ved 300 mg |

**F. Indirect effect estimates for SAEs.**

| ADA 160/80/40 |  |  |  |  |  |  |  |  |  |  |  |  |  |  |  |  |  |  |  |  |  |  |
| --- | --- | --- | --- | --- | --- | --- | --- | --- | --- | --- | --- | --- | --- | --- | --- | --- | --- | --- | --- | --- | --- | --- |
| 2.51 (0.25-25.37) | AJM 300 mg |  |  |  |  |  |  |  |  |  |  |  |  |  |  |  |  |  |  |  |  |  |
| 0.78 (0.13-4.65) | 0.31 (0.02-5.48) | Cob 250 mg |  |  |  |  |  |  |  |  |  |  |  |  |  |  |  |  |  |  |  |  |
| 0.19 (0.02-1.87) | 0.08 (0.00-1.88) | 0.25 (0.01-4.20) | Eld 10 mg/kg |  |  |  |  |  |  |  |  |  |  |  |  |  |  |  |  |  |  |  |
| 1.75 (0.48-6.45) | 0.70 (0.05-9.37) | 2.24 (0.26-18.95) | 8.98 (0.70-114.68) | Eld 25 mg/kg |  |  |  |  |  |  |  |  |  |  |  |  |  |  |  |  |  |  |
| 0.75 (0.33-1.70) | 0.30 (0.03-3.26) | 0.96 (0.15-6.27) | 3.83 (0.37-39.71) | 0.43 (0.10-1.79) | Etra 2 mg/kg |  |  |  |  |  |  |  |  |  |  |  |  |  |  |  |  |  |
| 0.66 (0.34-1.27) | 0.26 (0.03-2.73) | 0.84 (0.14-5.18) | 3.38 (0.34-33.24) | 0.38 (0.10-1.45) | 0.88 (0.36-2.16) | Etro 105 mg |  |  |  |  |  |  |  |  |  |  |  |  |  |  |  |  |
| 0.87 (0.39-1.96) | 0.35 (0.03-3.79) | 1.12 (0.17-7.28) | 4.48 (0.43-46.16) | 0.50 (0.12-2.07) | 1.17 (0.43-3.20) | 1.32 (0.55-3.18) | Fil 200 mg |  |  |  |  |  |  |  |  |  |  |  |  |  |  |  |
| 0.42 (0.19-0.93) | 0.17 (0.02-1.81) | 0.54 (0.08-3.47) | 2.15 (0.21-22.05) | 0.24 (0.06-0.99) | 0.56 (0.21-1.52) | 0.64 (0.27-1.51) | 0.48 (0.18-1.28) | Gol 2 mg/kg |  |  |  |  |  |  |  |  |  |  |  |  |  |  |
| 4.91 (0.56-43.12) | 1.96 (0.09-44.56) | 6.29 (0.40-98.61) | 25.19 (1.15-550.13) | 2.81 (0.24-33.15) | 6.58 (0.69-62.70) | 7.45 (0.83-67.15) | 5.63 (0.59-53.24) | 11.72 (1.24-110.46) | Gus 200 mg |  |  |  |  |  |  |  |  |  |  |  |  |  |
| 1.07 (0.57-2.02) | 0.43 (0.04-4.41) | 1.37 (0.22-8.35) | 5.49 (0.56-53.65) | 0.61 (0.16-2.33) | 1.43 (0.60-3.45) | 1.62 (0.99-2.67) | 1.23 (0.52-2.90) | 2.56 (1.10-5.96) | 0.22 (0.02-1.95) | Inf 5 mg/kg |  |  |  |  |  |  |  |  |  |  |  |  |
| 0.81 (0.10-6.29) | 0.32 (0.02-6.76) | 1.04 (0.07-14.78) | 4.16 (0.21-83.33) | 0.46 (0.04-4.91) | 1.09 (0.13-9.19) | 1.23 (0.15-9.81) | 0.93 (0.11-7.80) | 1.93 (0.23-16.18) | 0.16 (0.01-3.10) | 0.76 (0.10-6.00) | Iva 4 mg |  |  |  |  |  |  |  |  |  |  |  |
| 1.56 (0.75-3.27) | 0.62 (0.06-6.63) | 2.00 (0.32-12.66) | 8.02 (0.80-80.73) | 0.89 (0.22-3.57) | 2.09 (0.81-5.43) | 2.37 (1.05-5.34) | 1.79 (0.70-4.57) | 3.73 (1.48-9.41) | 0.32 (0.03-2.94) | 1.46 (0.66-3.23) | 1.93 (0.24-15.80) | Mir 300 mg |  |  |  |  |  |  |  |  |  |  |
| 3.24 (0.34-30.95) | 1.29 (0.05-31.19) | 4.15 (0.25-69.56) | 16.63 (0.72-385.38) | 1.85 (0.15-23.56) | 4.34 (0.42-44.86) | 4.92 (0.50-48.15) | 3.71 (0.36-38.10) | 7.74 (0.76-79.07) | 0.66 (0.03-14.37) | 3.03 (0.31-29.46) | 4.00 (0.20-79.92) | 2.07 (0.21-20.80) | Obe 50 mg |  |  |  |  |  |  |  |  |  |
| 0.37 (0.08-1.73) | 0.15 (0.01-2.26) | 0.48 (0.05-4.68) | 1.90 (0.13-27.68) | 0.21 (0.03-1.47) | 0.50 (0.10-2.60) | 0.56 (0.12-2.73) | 0.43 (0.08-2.20) | 0.89 (0.17-4.56) | 0.08 (0.01-1.02) | 0.35 (0.07-1.66) | 0.46 (0.04-5.59) | 0.24 (0.05-1.19) | 0.11 (0.01-1.66) | Oza 0.92 mg |  |  |  |  |  |  |  |  |
| 0.81 (0.54-1.21) | 0.32 (0.03-3.16) | 1.04 (0.18-5.90) | 4.16 (0.45-38.46) | 0.46 (0.13-1.60) | 1.09 (0.53-2.24) | 1.23 (0.73-2.08) | 0.93 (0.46-1.87) | 1.93 (0.97-3.85) | 0.16 (0.02-1.40) | 0.76 (0.46-1.24) | 1.00 (0.13-7.46) | 0.52 (0.28-0.96) | 0.25 (0.03-2.30) | 2.18 (0.49-9.66) | PBO |  |  |  |  |  |  |  |
| 3.24 (0.34-30.83) | 1.29 (0.05-31.11) | 4.15 (0.25-69.36) | 16.63 (0.72-384.35) | 1.85 (0.15-23.48) | 4.34 (0.42-44.70) | 4.92 (0.50-47.97) | 3.71 (0.36-37.97) | 7.74 (0.76-78.79) | 0.66 (0.03-14.33) | 3.03 (0.31-29.35) | 4.00 (0.20-79.70) | 2.07 (0.21-20.73) | 1.00 (0.04-23.04) | 8.73 (0.60-125.99) | 4.00 (0.44-36.70) | PF-00547659 22.5 mg |  |  |  |  |  |  |
| 1.32 (0.62-2.81) | 0.53 (0.05-5.63) | 1.69 (0.27-10.77) | 6.78 (0.67-68.61) | 0.75 (0.19-3.05) | 1.77 (0.67-4.65) | 2.00 (0.88-4.58) | 1.51 (0.59-3.91) | 3.15 (1.23-8.06) | 0.27 (0.03-2.50) | 1.23 (0.55-2.77) | 1.63 (0.20-13.43) | 0.85 (0.35-2.06) | 0.41 (0.04-4.11) | 3.56 (0.70-17.97) | 1.63 (0.86-3.09) | 0.41 (0.04-4.09) | Tofa 10 mg |  |  |  |  |  |
| 6.23 (0.72-53.75) | 2.49 (0.11-55.85) | 7.98 (0.52-123.37) | 31.97 (1.48-689.36) | 3.56 (0.31-41.41) | 8.35 (0.89-78.19) | 9.46 (1.07-83.71) | 7.14 (0.77-66.40) | 14.88 (1.61-137.76) | 1.27 (0.06-25.66) | 5.82 (0.66-51.21) | 7.69 (0.42-142.43) | 3.99 (0.44-36.21) | 1.92 (0.09-41.33) | 16.78 (1.26-223.13) | 7.69 (0.93-63.87) | 1.92 (0.09-41.21) | 4.72 (0.52-43.07) | Tul 1000/500 mg |  |  |  |  |
| 1.53 (0.74-3.18) | 0.61 (0.06-6.49) | 1.96 (0.31-12.38) | 7.87 (0.78-79.02) | 0.88 (0.22-3.49) | 2.06 (0.80-5.29) | 2.33 (1.04-5.20) | 1.76 (0.69-4.45) | 3.66 (1.46-9.17) | 0.31 (0.03-2.88) | 1.43 (0.65-3.14) | 1.89 (0.23-15.46) | 0.98 (0.41-2.34) | 0.47 (0.05-4.73) | 4.13 (0.83-20.62) | 1.89 (1.03-3.48) | 0.47 (0.05-4.71) | 1.16 (0.48-2.81) | 0.25 (0.03-2.23) | Upa 45 mg |  |  |  |
| 1.69 (0.88-3.27) | 0.68 (0.07-7.01) | 2.16 (0.35-13.30) | 8.68 (0.88-85.32) | 0.97 (0.25-3.72) | 2.27 (0.93-5.53) | 2.57 (1.22-5.38) | 1.94 (0.81-4.65) | 4.04 (1.70-9.58) | 0.34 (0.04-3.10) | 1.58 (0.77-3.25) | 2.09 (0.26-16.66) | 1.08 (0.48-2.44) | 0.52 (0.05-5.11) | 4.56 (0.94-22.06) | 2.09 (1.24-3.53) | 0.52 (0.05-5.09) | 1.28 (0.56-2.93) | 0.27 (0.03-2.40) | 1.10 (0.49-2.46) | Uste 6 mg/kg |  |  |
| 0.93 (0.30-2.92) | 0.37 (0.03-4.62) | 1.19 (0.16-9.19) | 4.79 (0.41-56.50) | 0.53 (0.10-2.74) | 1.25 (0.34-4.54) | 1.42 (0.43-4.65) | 1.07 (0.30-3.84) | 2.23 (0.63-7.94) | 0.19 (0.02-2.07) | 0.87 (0.27-2.83) | 1.15 (0.12-11.22) | 0.60 (0.17-2.06) | 0.29 (0.02-3.38) | 2.51 (0.40-15.70) | 1.15 (0.40-3.35) | 0.29 (0.02-3.37) | 0.71 (0.20-2.45) | 0.15 (0.01-1.60) | 0.61 (0.18-2.08) | 0.55 (0.17-1.81) | Ved 108 mg |  |
| 1.07 (0.74-1.54) | 0.43 (0.04-4.28) | 1.36 (0.23-8.04) | 5.47 (0.57-52.06) | 0.61 (0.17-2.22) | 1.43 (0.64-3.20) | 1.62 (0.86-3.05) | 1.22 (0.56-2.68) | 2.54 (1.17-5.52) | 0.22 (0.02-1.89) | 1.00 (0.54-1.84) | 1.32 (0.17-10.13) | 0.68 (0.33-1.40) | 0.33 (0.03-3.12) | 2.87 (0.62-13.26) | 1.32 (0.92-1.88) | 0.33 (0.03-3.11) | 0.81 (0.39-1.68) | 0.17 (0.02-1.46) | 0.69 (0.34-1.41) | 0.63 (0.33-1.19) | 1.14 (0.37-3.52) | Ved 300 mg |

**G. Indirect effect estimates for maintenance of clinical remission.**

| Etra 2 mg/kg |  | |  | |  | |  | | | | |
| --- | --- | --- | --- | --- | --- | --- | --- | --- | --- | --- | --- |
| 4.31 (1.14-16.29) | Etro 105 mg |  |  |  |  |  |  |  |  |  |  |
| 4.29 (1.08-17.11) | 0.99 (0.44-2.27) | Fil 200 mg |  |  |  |  |  |  |  |  |  |
| 4.61 (1.22-17.49) | 1.07 (0.68-1.68) | 1.08 (0.47-2.47) | Inf 5 mg/kg |  |  |  |  |  |  |  |  |
| 3.14 (0.84-11.79) | 0.73 (0.35-1.50) | 0.73 (0.32-1.66) | 0.68 (0.33-1.41) | Mir 200 mg |  |  |  |  |  |  |  |
| 4.21 (1.08-16.36) | 0.98 (0.45-2.13) | 0.98 (0.41-2.35) | 0.91 (0.42-2.01) | 1.34 (0.62-2.90) | Oza 0.92 mg |  |  |  |  |  |  |
| 9.58 (2.82-32.59) | 2.22 (1.32-3.73) | 2.23 (1.17-4.26) | 2.08 (1.23-3.52) | 3.05 (1.85-5.04) | 2.27 (1.27-4.08) | PBO |  |  |  |  |  |
| 4.68 (1.23-17.84) | 1.08 (0.51-2.29) | 1.09 (0.47-2.53) | 1.01 (0.48-2.16) | 1.49 (0.71-3.12) | 1.11 (0.50-2.47) | 0.49 (0.28-0.84) | Ris 180 mg |  | | | |
| 1.22 (0.30-4.89) | 0.28 (0.12-0.65) | 0.28 (0.11-0.71) | 0.26 (0.11-0.61) | 0.39 (0.17-0.89) | 0.29 (0.12-0.70) | 0.13 (0.07-0.25) | 0.26 (0.11-0.61) | Upa 30 mg |  | | |
| 3.89 (1.02-14.89) | 0.90 (0.42-1.92) | 0.91 (0.39-2.12) | 0.84 (0.39-1.81) | 1.24 (0.59-2.61) | 0.92 (0.41-2.06) | 0.41 (0.23-0.70) | 0.83 (0.38-1.80) | 3.19 (1.35-7.54) | Uste 90 mg |  | |
| 1.86 (0.41-8.46) | 0.43 (0.15-1.21) | 0.43 (0.14-1.30) | 0.40 (0.14-1.14) | 0.59 (0.21-1.65) | 0.44 (0.15-1.28) | 0.19 (0.08-0.47) | 0.40 (0.14-1.13) | 1.53 (0.50-4.64) | 0.48 (0.17-1.36) | Ved 108 mg |  |
| 2.68 (0.72-10.00) | 0.62 (0.31-1.26) | 0.62 (0.28-1.40) | 0.58 (0.28-1.19) | 0.85 (0.42-1.71) | 0.64 (0.30-1.36) | 0.28 (0.17-0.45) | 0.57 (0.28-1.19) | 2.20 (0.97-4.99) | 0.69 (0.33-1.43) | 1.44 (0.52-3.99) | Ved 300 mg |

**H. Indirect effect estimates for maintenance of endoscopic remission.**

| Etra 2 mg/kg |  | | | | | | |
| --- | --- | --- | --- | --- | --- | --- | --- |
| 1.30 (0.11-15.44) | Etro 105 mg |  | | | | | |
| 1.01 (0.04-23.69) | 0.78 (0.11- 5.50) | Inf 5 mg/kg |  | |  | | |
| 1.31 (0.11-15.14) | 1.01 (0.06-16.63) | 1.30 (0.04-39.54) | Oza 0.92 mg |  |  |  |  |
| 2.72 (0.63-11.69) | 2.09 (0.28-15.43) | 2.69 (0.16-44.05) | 2.07 (0.29-14.78) | PBO |  |  |  |
| 1.19 (0.10-13.65) | 0.92 (0.06-15.00) | 1.18 (0.04-35.71) | 0.91 (0.06-14.51) | 0.44 (0.06- 3.09) | Ris 180 mg |  |  |
| 0.29 (0.02- 3.44) | 0.22 (0.01- 3.77) | 0.29 (0.01- 8.93) | 0.22 (0.01- 3.65) | 0.11 (0.01- 0.78) | 0.25 (0.02- 3.99) | Upa 30 mg |  |
| 1.06 (0.09-12.21) | 0.82 (0.05-13.41) | 1.05 (0.03-31.93) | 0.81 (0.05-12.98) | 0.39 (0.06- 2.77) | 0.89 (0.06-14.19) | 3.63 (0.22-59.13) | Uste 90 mg |

**I. Indirect effect estimates for quality of life.**

| Cob 250 mg |  |  | |  |  | |
| --- | --- | --- | --- | --- | --- | --- |
| 1.23 (0.30-5.12) | Eld 25 mg/kg |  |  |  |  |  |
| 0.94 (0.25-3.54) | 0.77 (0.39-1.52) | Fil 200 mg |  |  |  |  |
| 2.05 (0.26-16.39) | 1.66 (0.29-9.52) | 2.17 (0.41-11.46) | Gol 2 mg/kg |  |  |  |
| 0.70 (0.17-2.85) | 0.56 (0.24-1.31) | 0.74 (0.38-1.42) | 0.34 (0.06-1.92) | Guse 200 mg |  |  |
| 2.11 (0.58-7.62) | 1.71 (0.93-3.16) | 2.24 (1.64-3.04) | 1.03 (0.20-5.27) | 3.04 (1.70-5.44) | PBO |  |
| 1.10 (0.29-4.25) | 0.89 (0.43-1.88) | 1.17 (0.69-1.96) | 0.54 (0.10-2.90) | 1.59 (0.77-3.25) | 0.52 (0.34-0.79) | Upa 45 mg |

**Drugs defined in columns are compared to those defined in rows. The estimates in the cells are** **odd ratios (ORs), with 95%** **confidence intervals (CI). The upper left box shows treatment with ORs larger than 1.0 for efficacy outcomes.**

**PBO: Placebo; ADA 160/80/40 group: ADA 160/80/40; Cobitolimod 250 mg: Cob 250 mg; Eldelumab 25 mg/kg: Eld 25 mg/kg; Eldelumab 10 mg/kg: Eld 10 mg/kg; Etrasimod 2 mg/kg : Etra 2 mg/kg ; Etrolizumab 105 mg: Etro 105 mg; Filgotinib 200 mg: Fil 200 mg; Golimumab 2 mg/kg: Gol 2 mg/kg; Guselkumab 200 mg : Gus 200 mg ; Infliximab 5 mg/kg: Inf 5 mg/kg; Obefazimod 50 mg: Obe 50 mg; Upadacitinib 45 mg : Upa 45 mg ; Vedolizumab 108 mg: Ved 108 mg; Vedolizumab 300 mg: Ved 300 mg; Ivarmacitinib 4 mg: Iva 4 mg; Ozanimod 0.92 mg: Oza 0.92 mg; Tofacitinib 10 mg: Tofa 10 mg; Ustekinumab 6 mg/kg: Uste 6 mg/kg; Mirikizumab 300 mg: Mir 300 mg; Risankizumab 1200 mg: Ris 1200 mg; Daclizumab 2 mg/kg: Dac 2 mg/kg; Tulisokibart 1000/500 mg: Tul1000/500 mg; PF-00547659 22.5 mg: PF-00547659 22.5 mg; AJM 300 960 mg: AJM 300 960 mg**

**Table S4. SUCRA values and ranks of treatments.**

| Treatment | Maintenance of clinical remission | | Maintenance of Endoscopic remission | |
| --- | --- | --- | --- | --- |
|  | SUCRA value (%) | Rank | SUCRA value (%) | Rank |
| Placebo | 6.15 | 12 | 19.49 | 8 |
| Etrolizumab 105 mg | 38.45 | 9 | 45.08 | 7 |
| Infliximab 5mg/kg | 36.02 | 10 | 52.77 | 3 |
| Upadacitinib 30 mg | 84.53 | 2 | 82.11 | 1 |
| Vedolizumab 300 mg | 62.17 | 4 | / | / |
| Filgotinib 200 mg | 39.18 | 8 | / | / |
| Etrasimod 2 mg/kg | 88.27 | 1 | 55.34 | 2 |
| Vedolizumab 108 mg | 73.23 | 3 | / | / |
| Ozanimod 0.92mg | 40.28 | 7 | 45.53 | 6 |
| Ustekinumab 90mg | 43.37 | 6 | 51.55 | 4 |
| Mirikizumab 200mg | 53.90 | 5 | / | / |
| Risankizumab 180mg | 34.45 | 11 | 48.13 | 5 |

1. **Quality of life**

| Treatment | Quality of life | |
| --- | --- | --- |
|  | SUCRA value (%) | Rank |
| Placebo | 18.81 | 7 |
| Cobitolimod 250 mg | 59.28 | 3 |
| Eldelumab 25 mg/kg | 48.79 | 5 |
| Filgotinib 200 mg | 61.93 | 2 |
| Golimumab 2 mg/kg | 31.70 | 6 |
| Guselkumab 200 mg | 75.66 | 1 |
| Upadacitinib 45 mg | 53.84 | 4 |

**Figure S1. Risk of bias summary.**

**A**

**
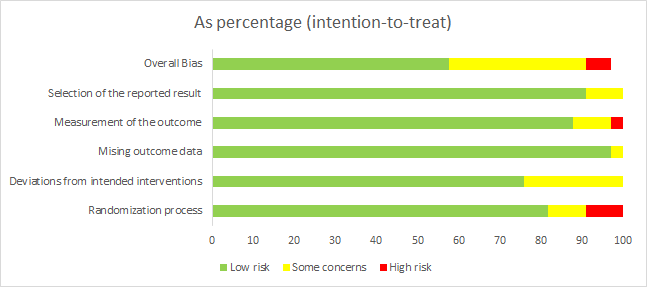
**

**B**

**
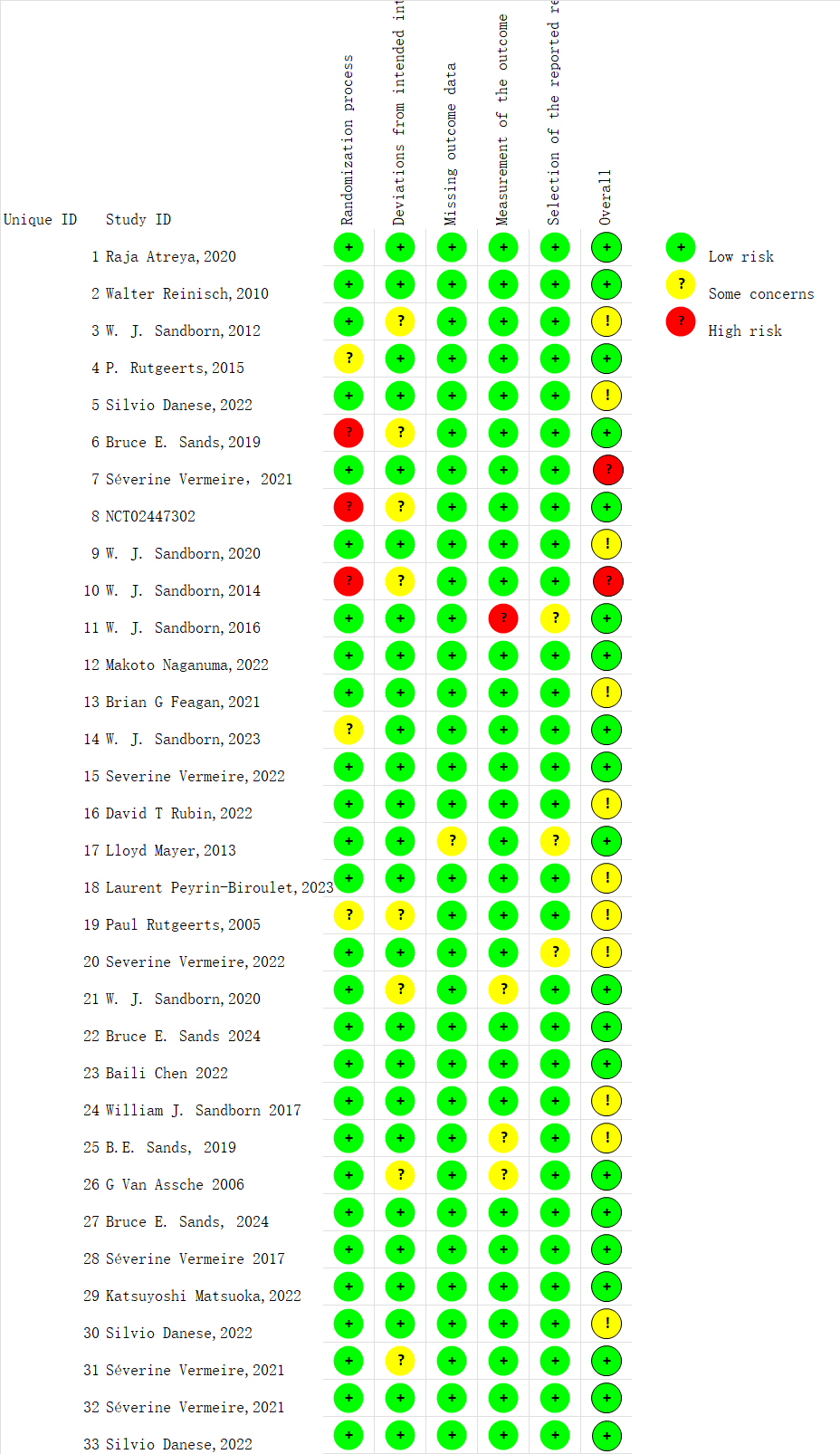
**

**Figure S2. Network of included trials in moderate to severe ulcerative colitis patients.**

**A. Network of Clinical remission in the induction phase.**


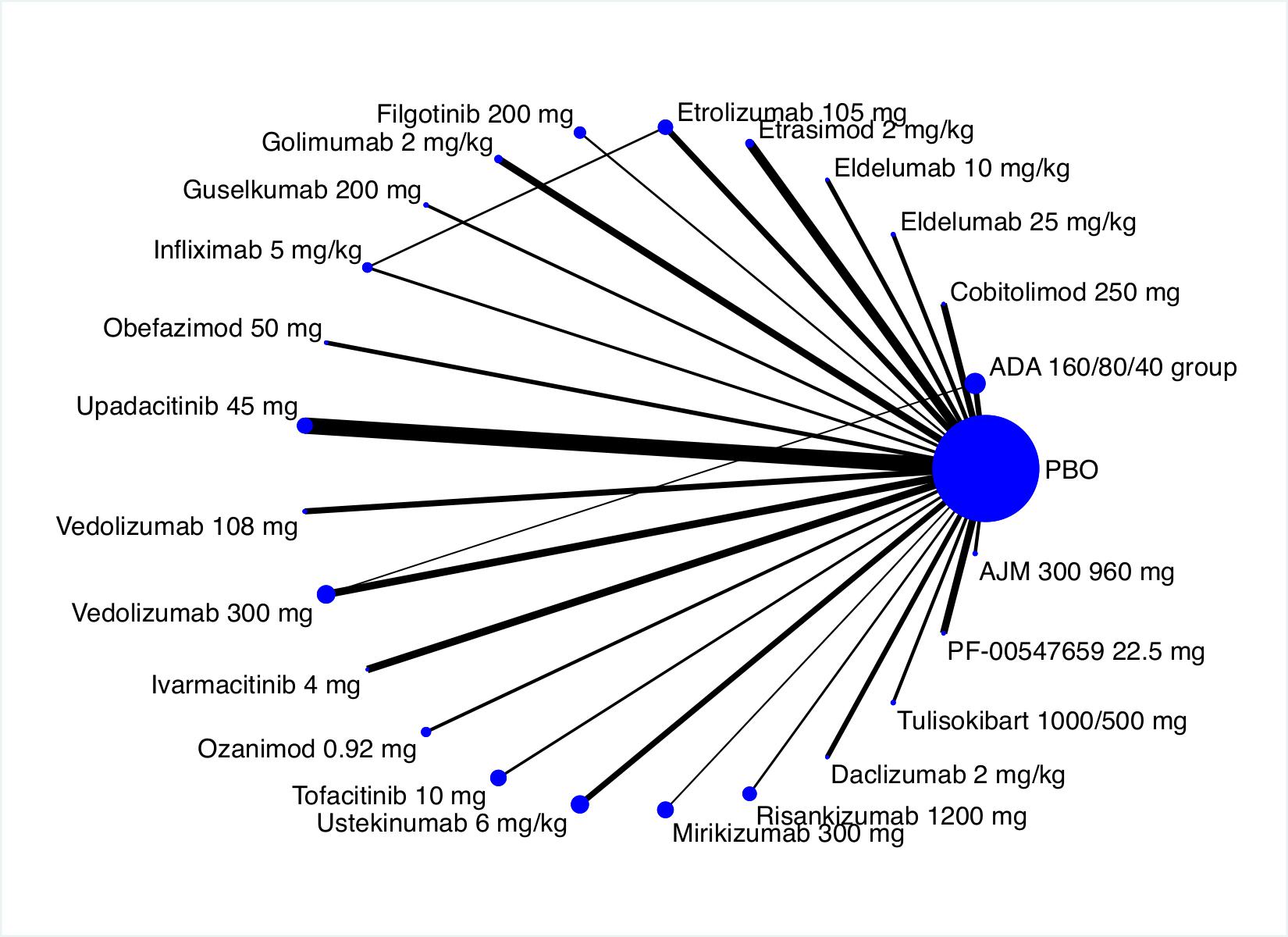


**B. Network of Clinical response in the induction phase.**

**
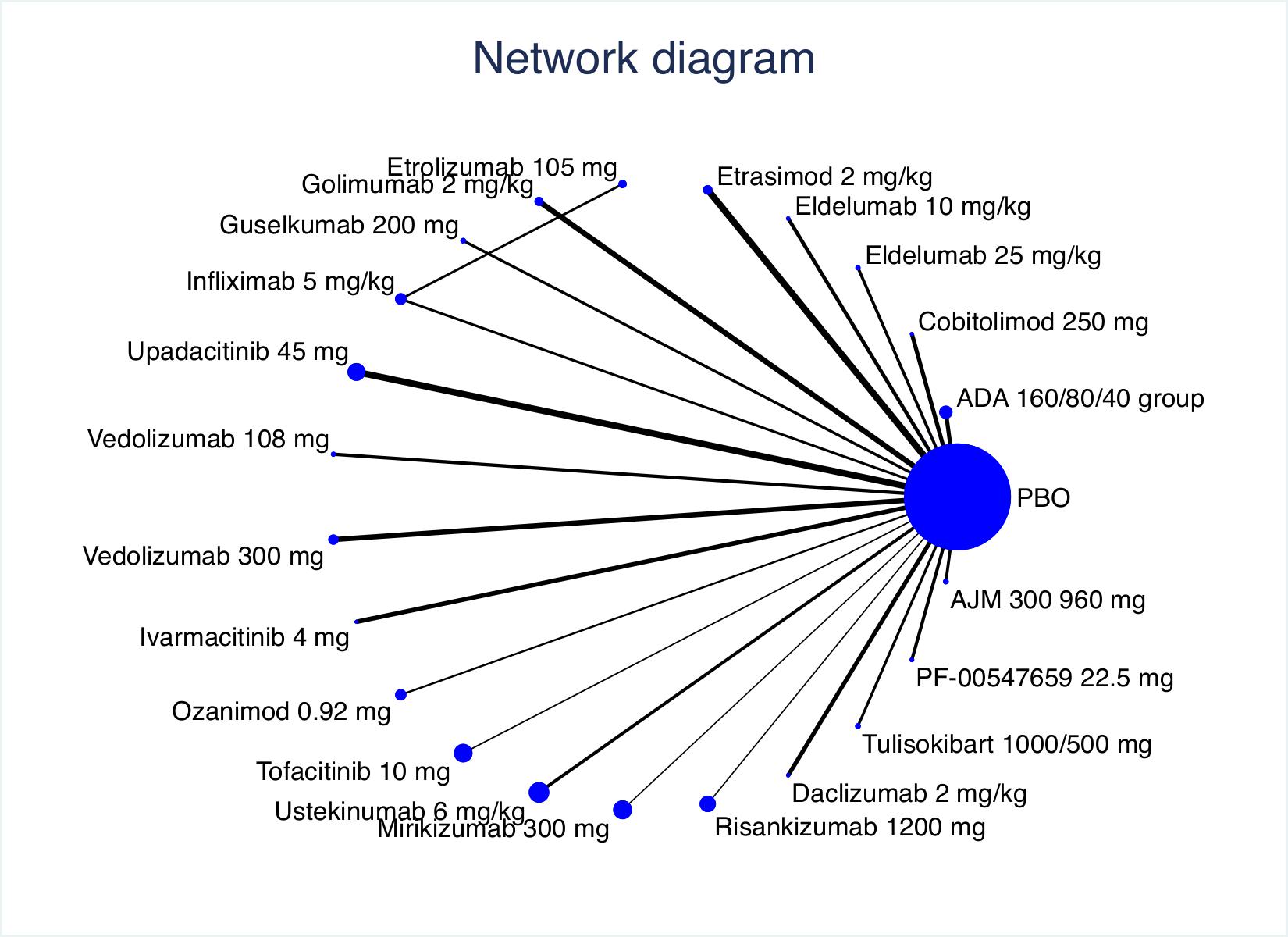
**

**C. Network of Endoscopic remission in the induction phase.**

**
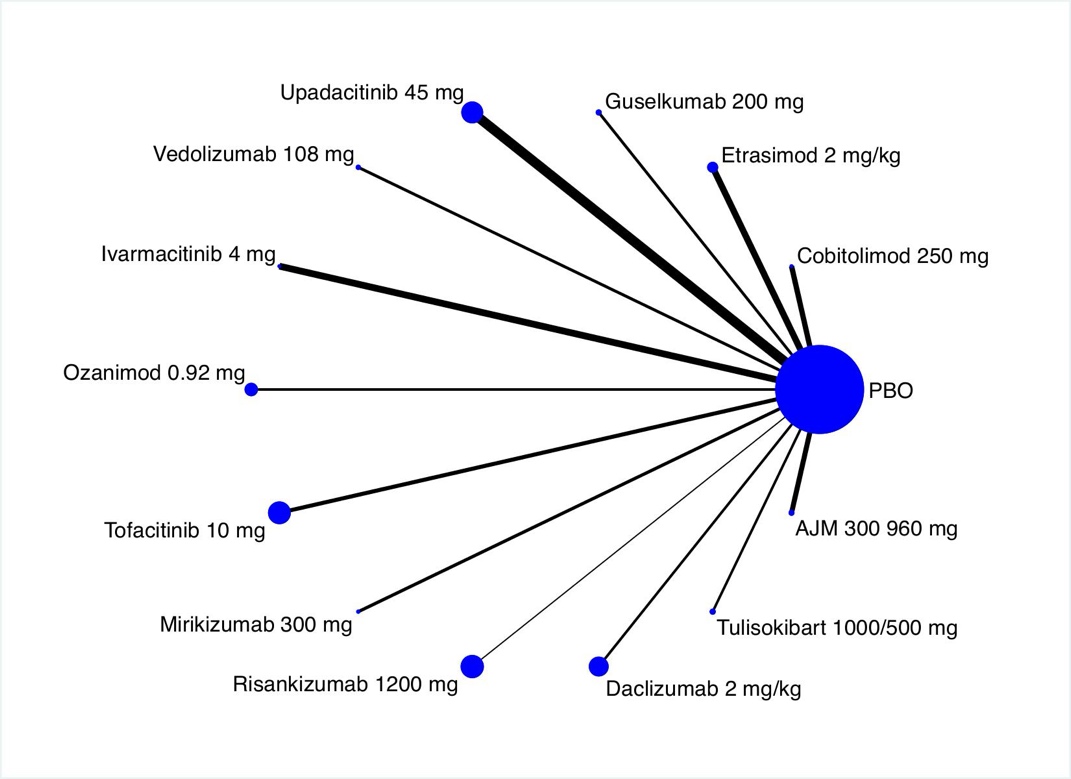
**

**D. Network of Mucosal healing in the induction phase.**

**
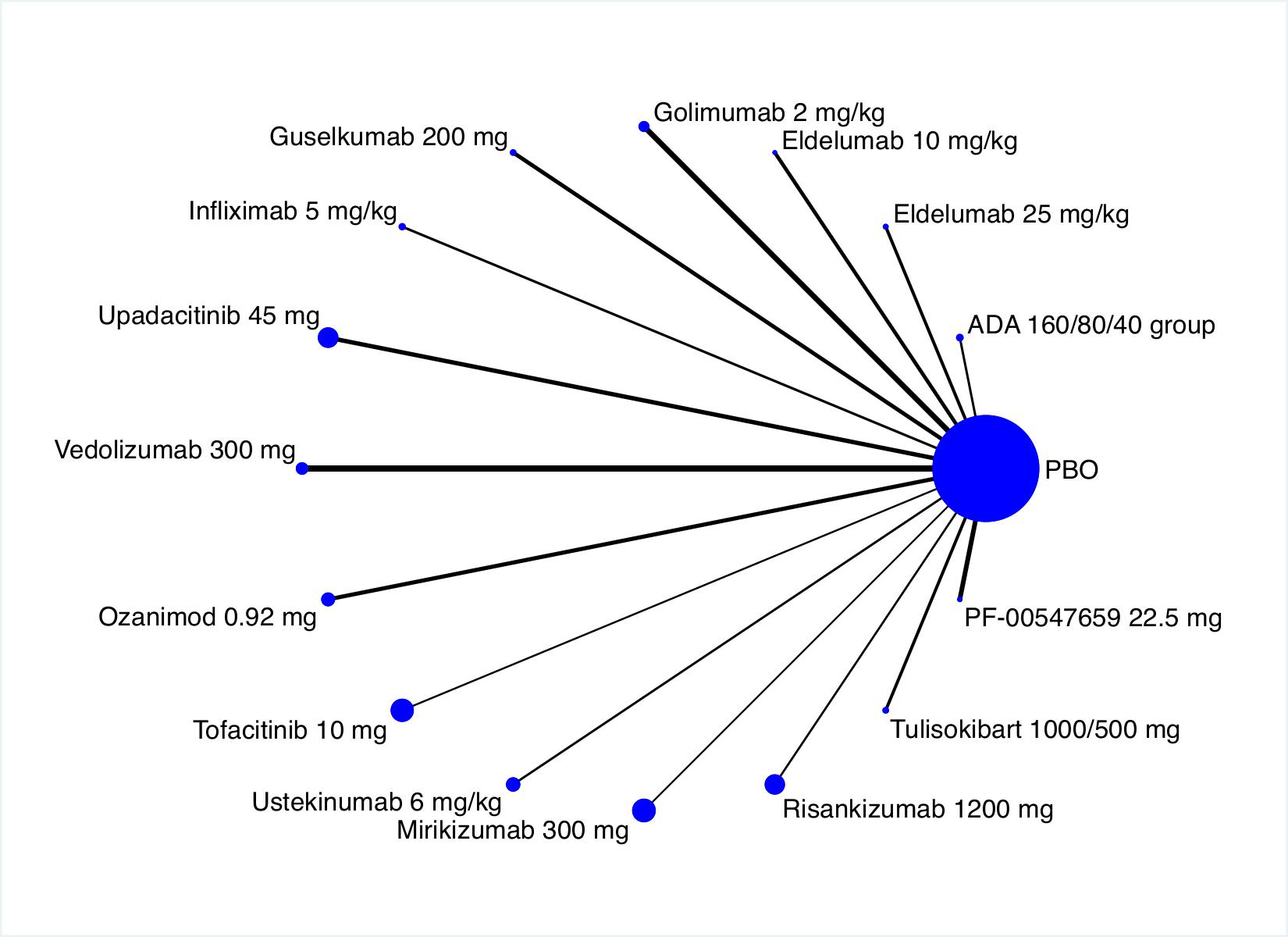
**

**E. Network of AEs.**


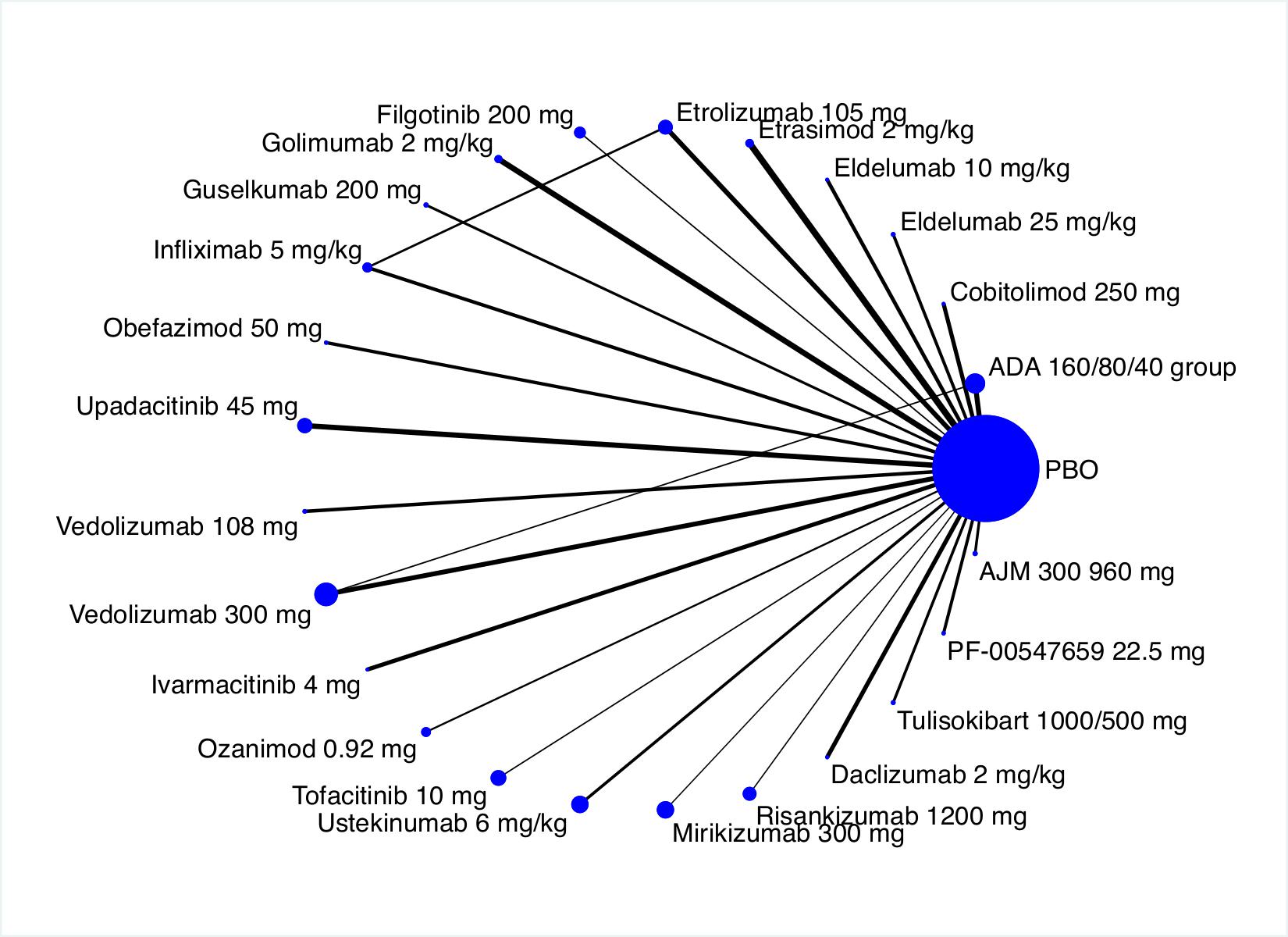


**F. Network of SAEs.**


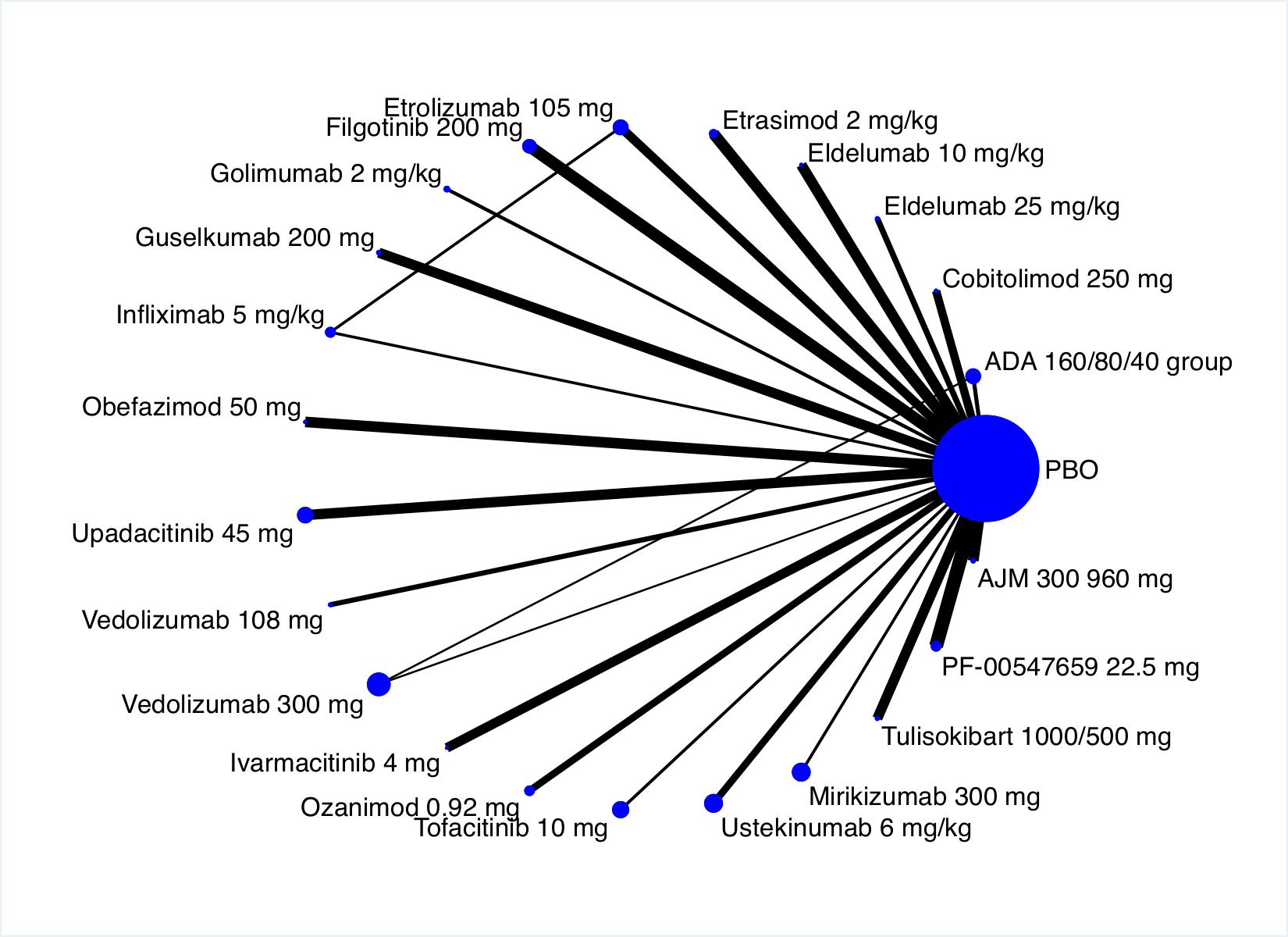


**G. Network of Clinical remission in the maintenance phase.**

**
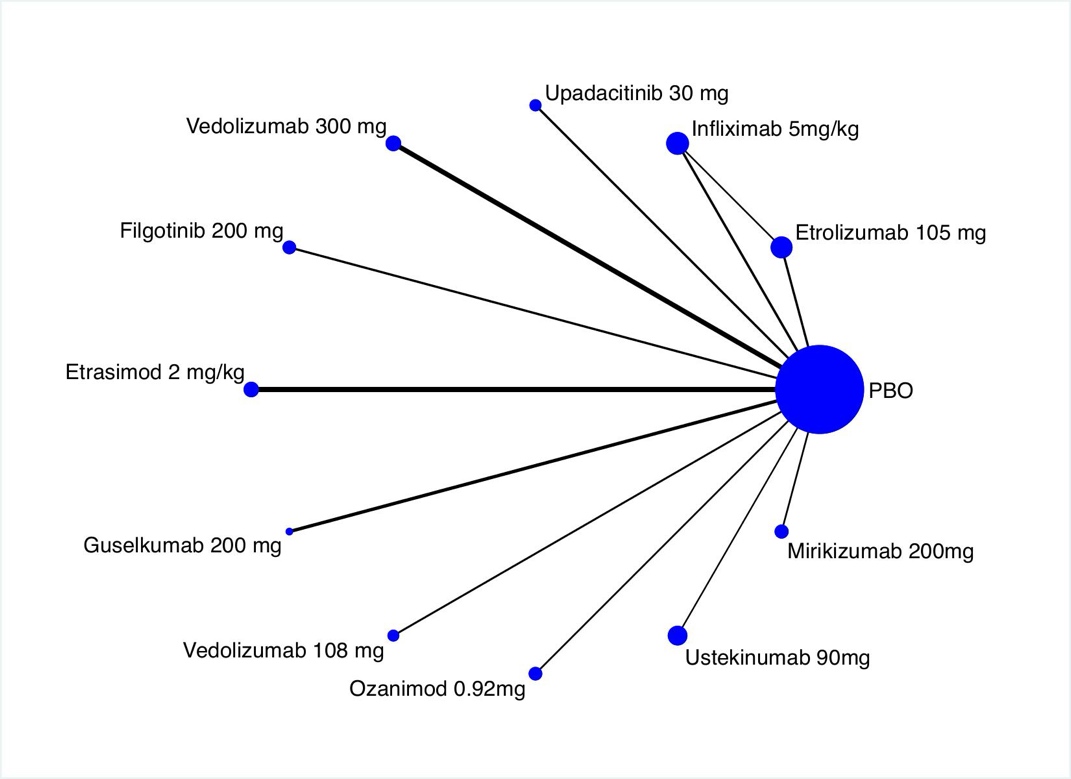
**

**H. Network of Endoscopic remission in the maintenance phase.**

**
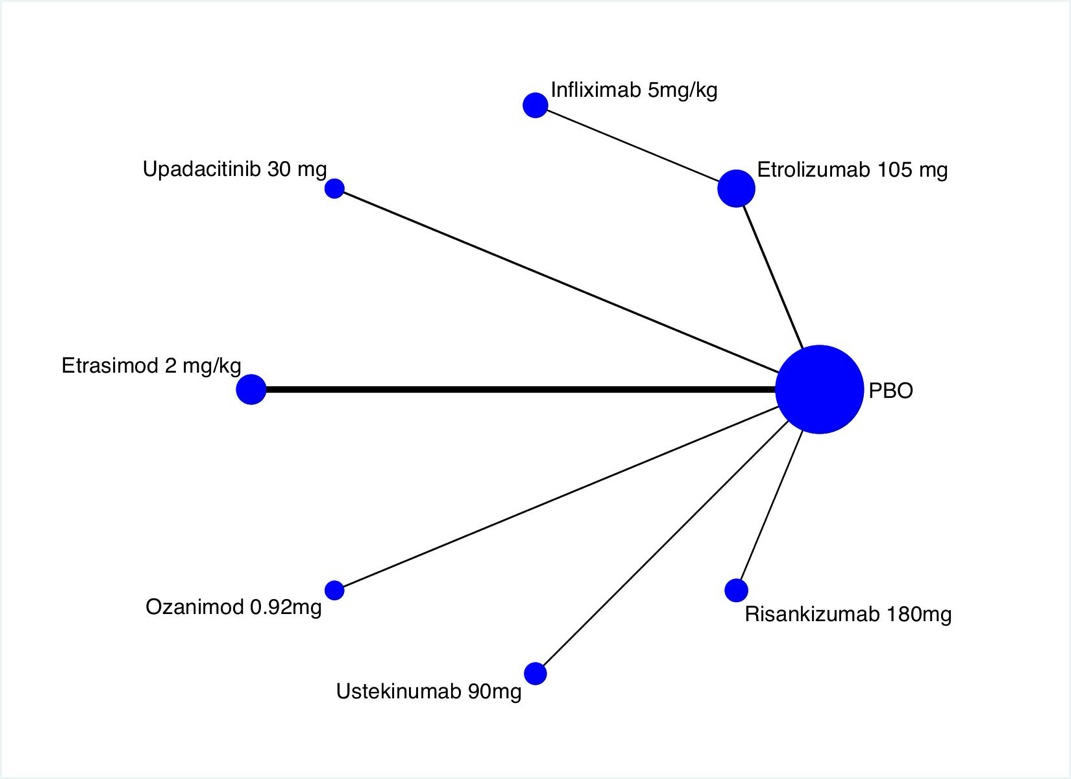
**

**I. Network of Quality of life.**


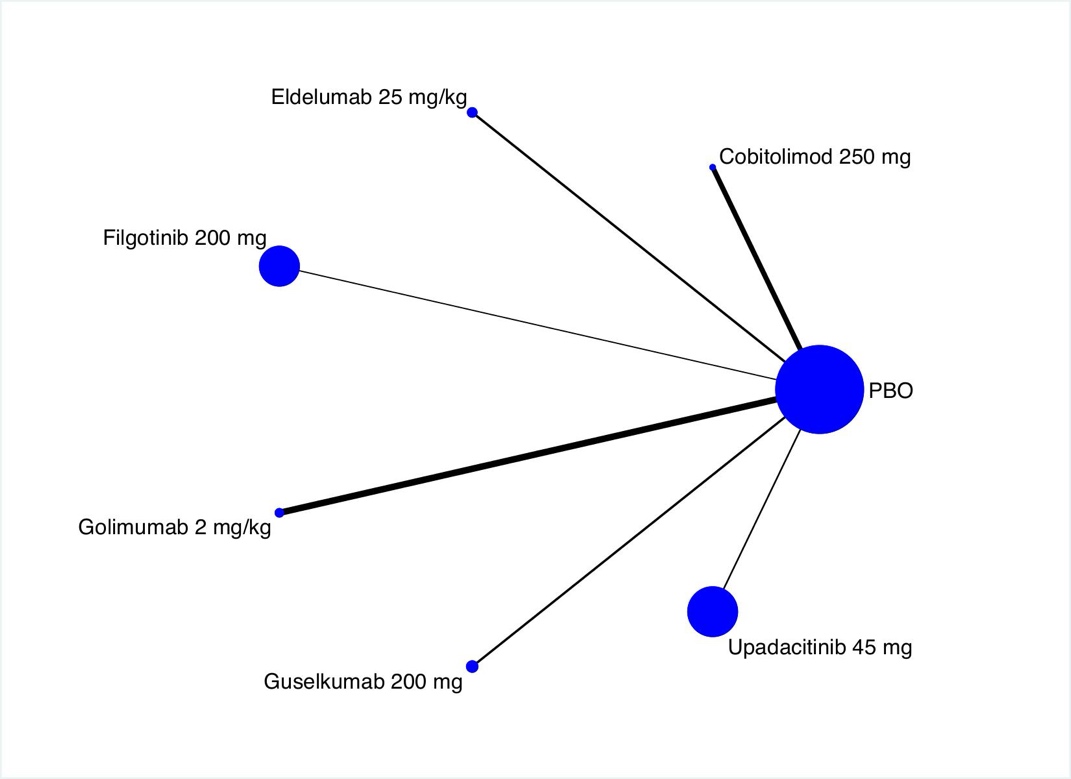


**PBO: Placebo**

**The size of the nodes and the thickness of the edges are weighted according to the number of studies evaluating each treatment and direct comparison respectively.**

**Figure S3. Efficacy outcomes (i.e., clinical remission and endoscopic remission) of targeted therapies in moderate to severe ulcerative colitis patients for the maintenance phase****:** **forest plot.**

1. **Clinical remission**

**
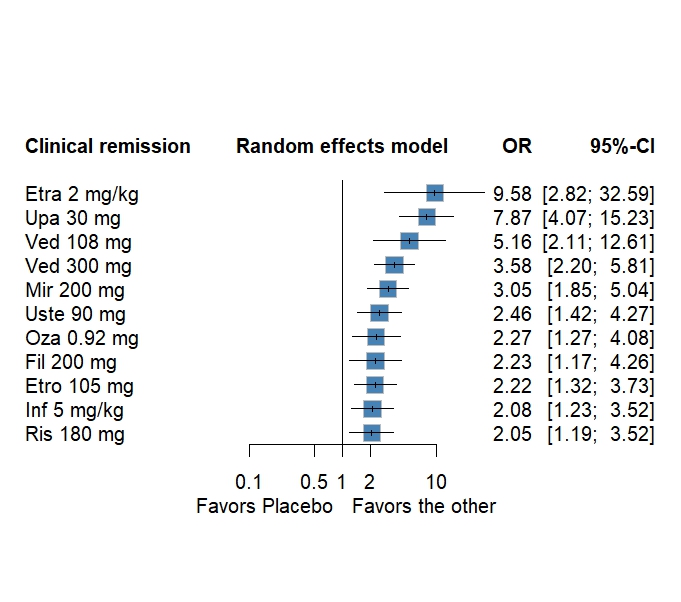
**

1. **Endoscopic remission**

**
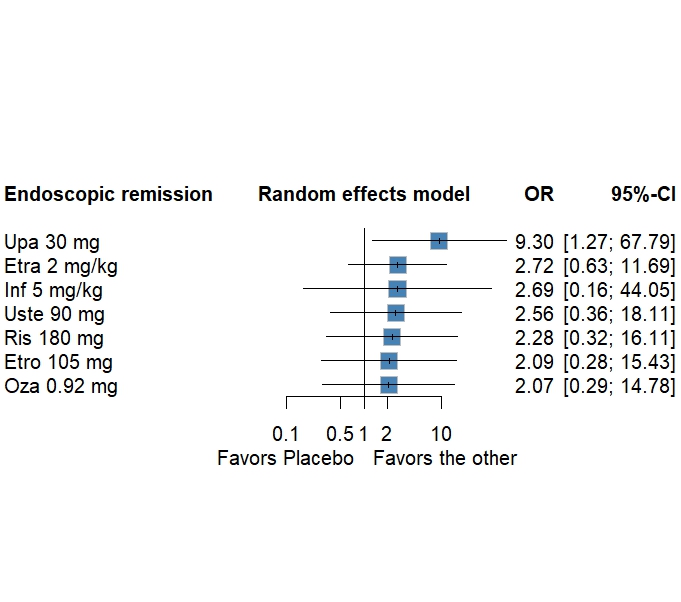
**

**Tul 1000/500 mg: Tulisokibart 1000/500 mg; Etra 2 mg/kg: Etrasimod 2 mg/kg; Upa 30 mg: Upadacitinib 30 mg; Etro 105 mg: Etrolizumab 105 mg; Inf 5 mg/kg: Infliximab 5 mg/kg; Ved 300 mg: Vedolizumab 300 mg; Fil 200 mg: Filgotinib 200 mg; Guse 200 mg: Guselkumab 200 mg; Ved 108 mg: Vedolizumab 108 mg; Oza 0.92 mg: Ozanimod 0.92mg; Uste 90 mg: Ustekinumab 90mg; Mir 200 mg: Mirikizumab 200mg; Ris 180 mg: Risankizumab 180mg**

**Cl:** **confidence intervals**

**Figure S4. Efficacy of targeted therapies in moderate to severe ulcerative colitis patients of quality of life:** **forest plot.**

**
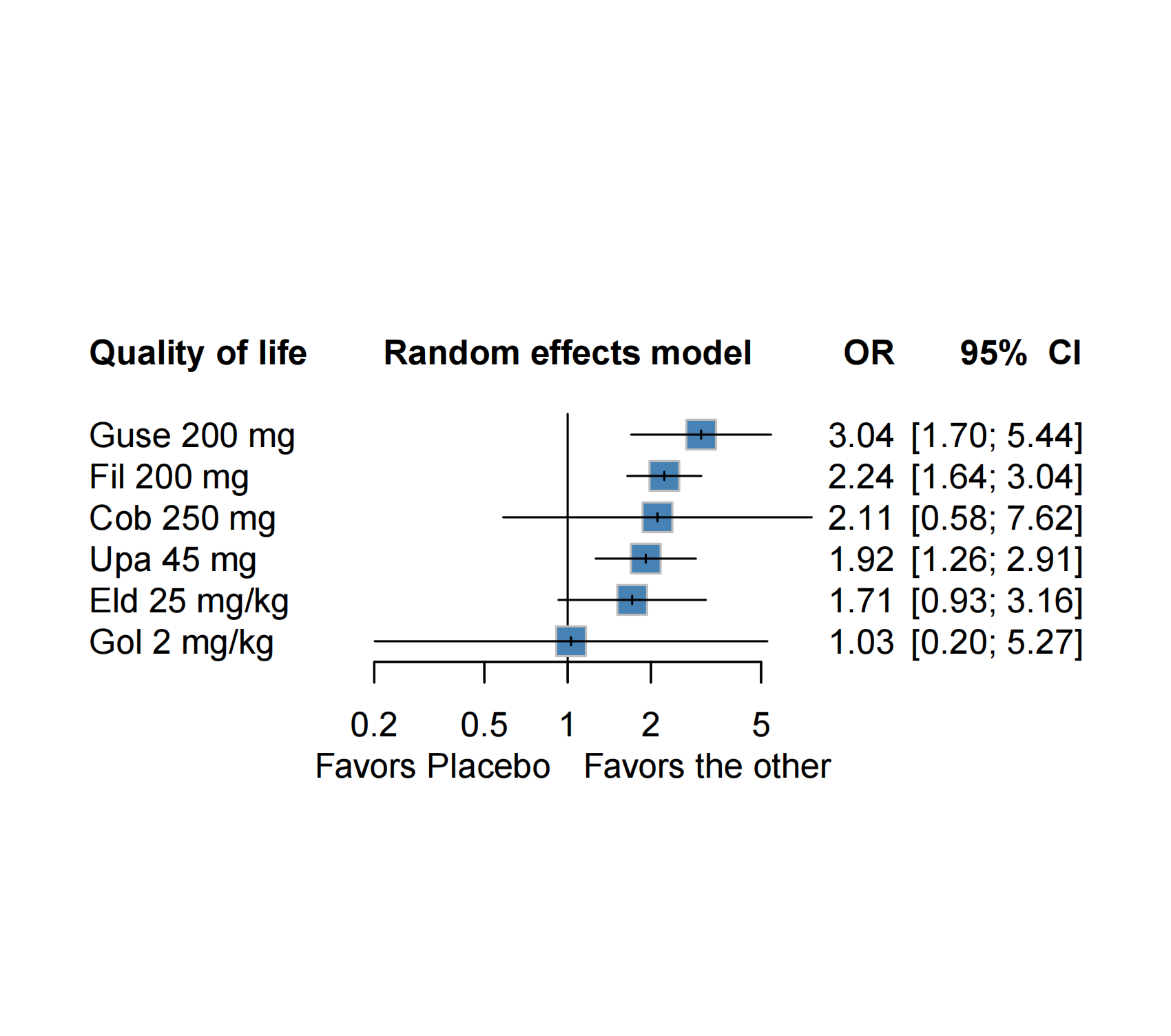
**

**Guse 200 mg: Guselkumab 200 mg; Fil 200 mg: Filgotinib 200 mg; Cob 250 mg: Cobitolimod 250 mg; Upa 45 mg: Upadacitinib 45 mg； Eld 25 mg/kg: Eldelumab 25 mg/kg； Gol 2 mg/kg: Golimumab 2 mg/kg**

**Cl:** **confidence intervals**

**Figure S5. Funnel plots for different outcomes.**

**A. Clinical remission in the induction phase**

**
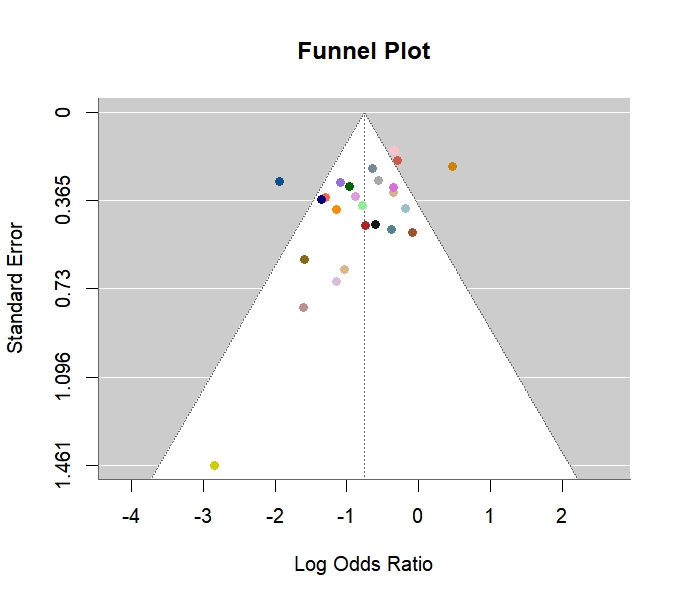
**

**B. Clinical response in the induction phase**

**C. Endoscopic remission in the induction phase**

**
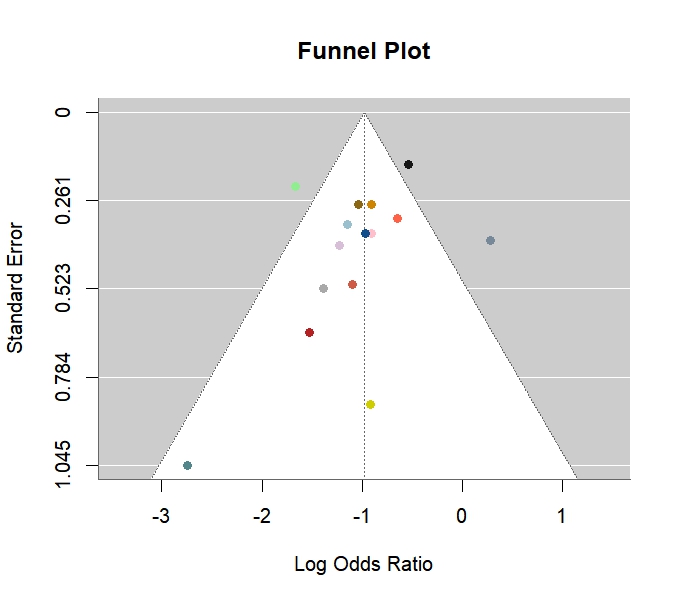
**

**D. Mucosal healing in the induction phase**

**
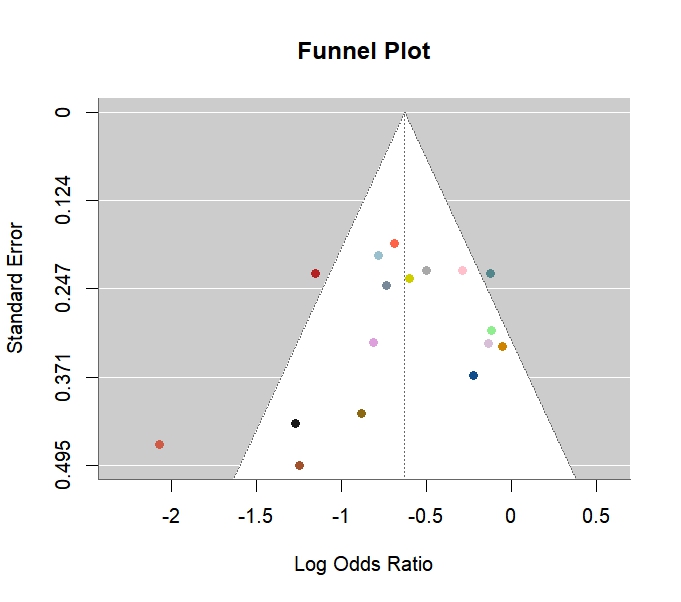
**

**E. Adverse events**

**
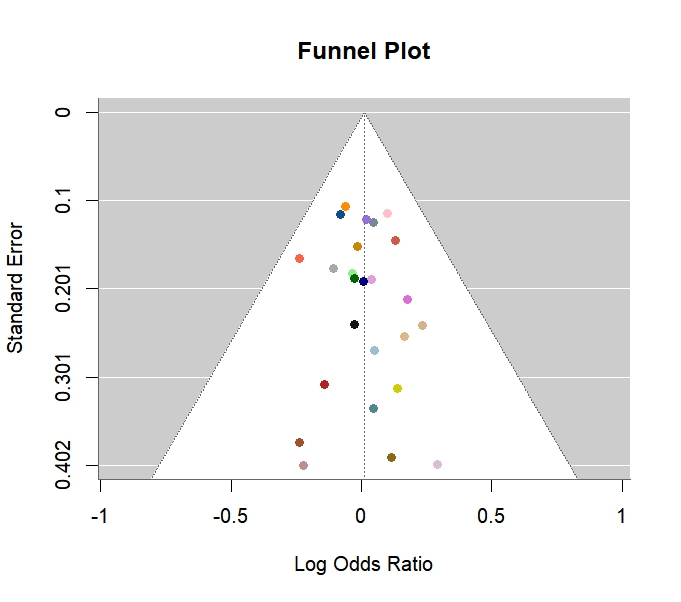
**

**F. Serious adverse events**

**
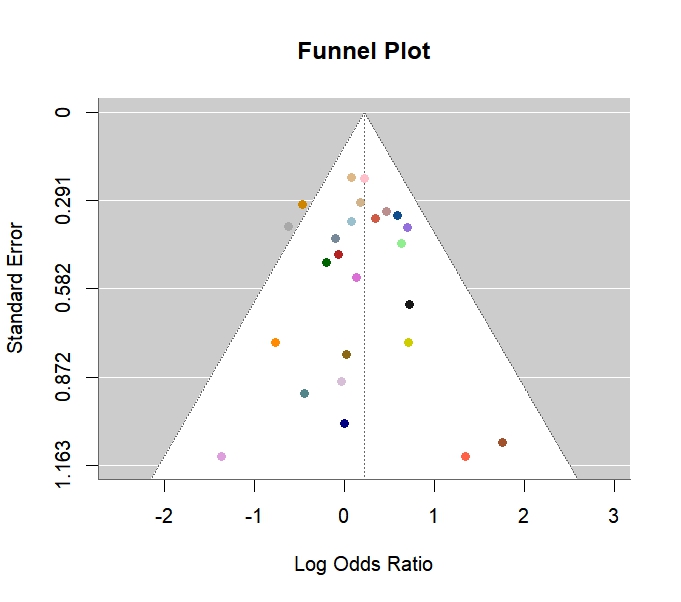
**

**G. Clinical remission in the maintenance phases**

**
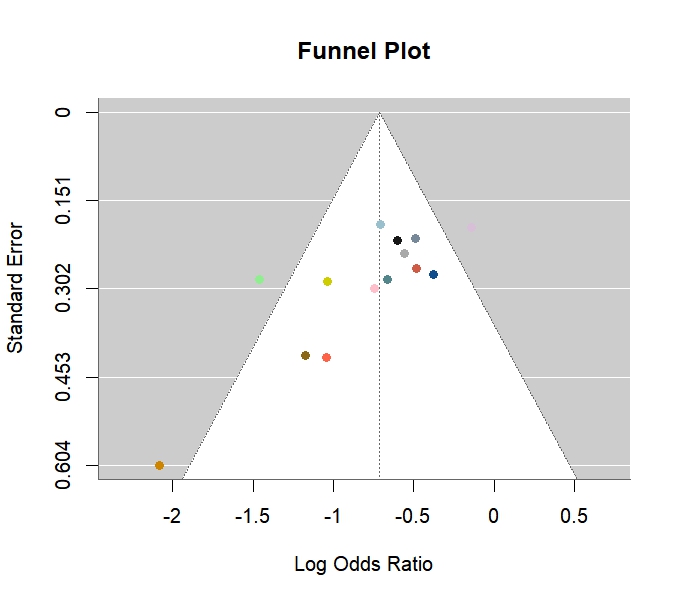
**

**H. Endoscopic remission in the maintenance phases**

**
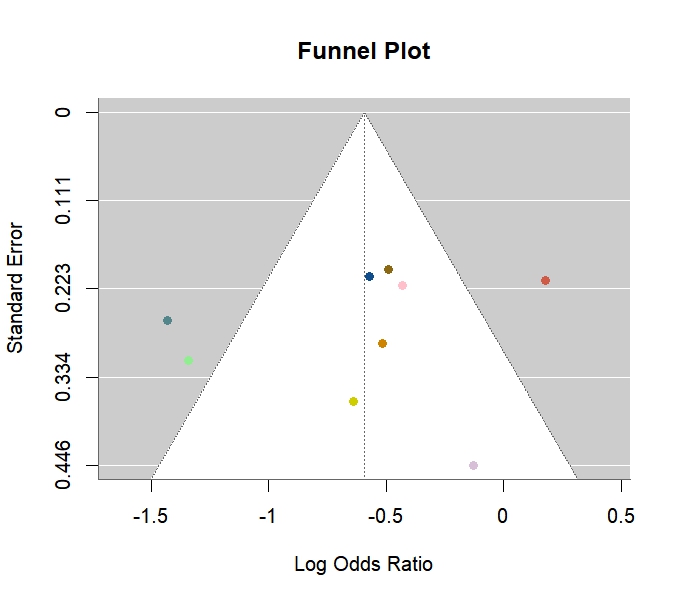
**

**I. Quality of life**

**
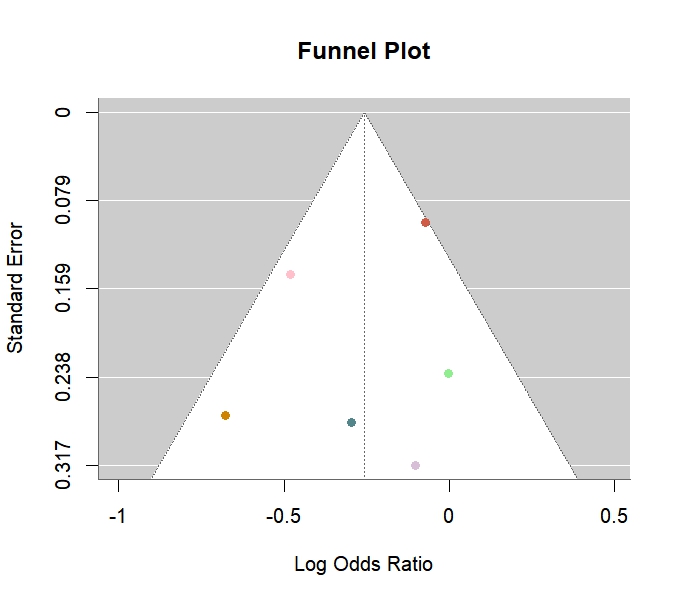
**
